# Biophysical Studies of the Membrane-Embedded and Cytoplasmic Forms of the Glucose-Specific Enzyme II of the *E. coli* Phosphotransferase System (PTS)

**DOI:** 10.1371/journal.pone.0024088

**Published:** 2011-09-15

**Authors:** Mohammad Aboulwafa, Milton H. Saier

**Affiliations:** Molecular Biology Department, University of California at San Diego, La Jolla, California, United States of America; University of Arkansas for Medical Sciences, United States of America

## Abstract

The glucose Enzyme II transporter complex of the *Escherichia coli* phosphotransferase system (PTS) exists in at least two physically distinct forms: a membrane-integrated dimeric form, and a cytoplasmic monomeric form, but little is known about the physical states of these enzyme forms. Six approaches were used to evaluate protein-protein and protein-lipid interactions in this system. Fluorescence energy transfer (FRET) using MBP-II^Glc^-YFP and MBP-II^Glc^-CFP revealed that the homodimeric Enzyme II complex in cell membranes is stable (FRET^-^) but can be dissociated and reassociated to the heterodimer only in the presence of Triton X100 (FRET^+^). The monomeric species could form a heterodimeric species (FRET^+^) by incubation and purification without detergent exposure. Formaldehyde cross linking studies, conducted both *in vivo* and *in vitro*, revealed that the dimeric MBP-II^Glc^ activity decreased dramatically with increasing formaldehyde concentrations due to both aggregation and activity loss, but that the monomeric MBP-II^Glc^ retained activity more effectively in response to the same formaldehyde treatments, and little or no aggregation was observed. Electron microscopy of MBP-II^Glc^ indicated that the dimeric form is larger than the monomeric form. Dynamic light scattering confirmed this conclusion and provided quantitation. NMR analyses provided strong evidence that the dimeric form is present primarily in a lipid bilayer while the monomeric form is present as micelles. Finally, lipid analyses of the different fractions revealed that the three lipid species (PE, PG and CL) are present in all fractions, but the monomeric micellar structure contains a higher percentage of anionic lipids (PG & CL) while the dimeric bilayer form has a higher percentage of zwitterion lipids (PE). Additionally, evidence for a minor dimeric micellar species, possibly an intermediate between the monomeric micellar and the dimeric bilayer forms, is presented. These results provide convincing evidence for interconvertible physical forms of Enzyme-II^Glc^.

## Introduction

In previous publications, we identified and partially characterized a novel form of the Enzyme II complexes of the bacterial phosphotransferase system, PTS, which both transports and phosphorylates its substrates in a single phosphoenol pyruvate (PEP)-dependent step. The novel form of these enzyme complexes proved to be localized to the cytoplasm of the *E. coli* cell and differed in properties from the previously characterized and more abundant membrane integrated complexes [1]. The membrane-integrated form proved to be dimeric while the soluble form was monomeric [2]. These two fractions could be easily separated by gel filtration where Peak 1 (the dimer) appeared in the void volume while Peak 2 (the monomer) was included.

All PTS Enzymes II have been shown to catalyze two vectorial reactions, the unidirectional PEP-dependent sugar phosphorylation reaction and a bidirectional transphosphorylation reaction in which the phosphoryl motiety of an intracellular sugar phosphate is transferred to an incoming radioactive sugar present initially extracytoplasmically [3], [4]. In this reaction, uptake of the radioactive sugar is coupled to the export of the sugar motiety of the intracellular sugar-phosphate in intact cells [4]. The PTS can phosphorylate sugars in both vectorial and non-vectorial reactions [5], [6]. This fact provides assays for the Enzyme II complexes not available for most transport systems.

The glucose Enzyme II complex (II^Glc^) has been studied in greatest detail and has been purified using both his-tagged and maltose-binding protein-fused constructs [1], [2], [7], [8]. The purified protein also exists in the two states (soluble and membrane integrated), and *in vitro* conditions that allow the interconversion of these two forms have been identified. The same appears to be true for all of the PTS Enzyme II complexes, and possibly for other *E. coli* transporters as well [1], [2], [9].

In this paper, we use physical methods to characterize and contrast the soluble versus membrane forms of the glucose Enzyme II complex. Approaches used include Fluorescence Resonance Energy Transfer (FRET) [10], [11], [12], formaldehyde crosslinking [13], [14], cryoelectron microscopy [15], [16], Dynamic Light Scattering (D.L.S.) [17], [18] and NMR spectroscopy correlated with catalytic activity measurements [1], [2], [19]. We also compare the phospholipid (PL) contents (phosphatidyl ethanolamine (PE), phosphatidyl glycerol (PG) and cardiolipin (CL)), showing that the soluble forms of II^Glc^ have a higher anionic PL content than the membranes. The results clearly suggest that the soluble II^Glc^ is present in a micellar structure, and that there are intermediate states between the monomeric micellar structure and the fully membrane integrated dimeric bilayer structure, one of which appears to be a larger dimeric micelle.

## Methods

### *In vitro* FRET analyses

Protein-protein interactions of II^Glc^ was studied by FRET analysis. The expressed II^Glc^ was fused at its N-terminus to MBP and at its C-terminus to either CFP or YFP as the fluorophore.

#### Construction of pMALE-*ptsG-cfp* and pMALE-*ptsG-yfp* recombinant plasmids

Standard methods were used for DNA amplification, restriction enzyme digestion, agarose gel electrophoresis, ligation, and transformation [20].

#### Cloning of *ptsG* into the pMAL^TM^-c2X plasmid

*ptsG* was amplified using a pair of primers that generate an *Eco*RI restriction site at the beginning of the gene and a *Bam*HI site at the end of the gene while removing the stop codon. The forward primer sequence was 5′-cggaattcatgtttaagaatgcatttgcta-3′ while the reverse primer sequence was 5′-cgggatccgtggttacggatgtactcatccat-3′. The recombinant plasmid pMALE-*ptsG* was used as a template. The PCR product was electrophoresed, and the band of proper size was cut out and purified using a gel extraction kit (Biomiga, USA). The PCR product was digested with and ligated into the *Eco*RI/*Bam*HI sites of the pMAL^TM^-c2X plasmid. The ligated mixture was transformed into competent *E. coli* DH5α cells, where colonies were obtained. Ten colonies were picked, purified onto LB/100 µg/ml ampicillin and cultured in 5 ml aliquots of LB/100 µg/ml ampicillin for plasmid extraction. For verification of the right clone, the plasmid preps were digested with *Eco*RI/*Bam*HI using 5 µl aliquots to detect a band on agarose of size equivalent to the *ptsG* gene. The insert within the plasmid that showed the expected *ptsG* size was subjected to DNA sequencing using three primers; the first (forward) was designed near the end of *malE* and was of the sequence 5′-cagcggtcgtcagactgtcgatg-3′, the second primer (forward) was designed within the *ptsG* gene and had the sequence 5′-accgtattaagctgcctgagtatc-3′ while the third one (reverse) was within pMAL^TM^-c2X downstream the insert and has the sequence 5′-cattcgccattcaggctgcgcaac-3′.

#### Cloning of *cfp* and *yfp* into the recombinant plasmid pMALE-*ptsG*

*cfp* and *yfp* plasmids were separately extracted from *E. coli* DH5α harboring these plasmids as follows. The host strains were grown in 5 ml aliquots of LB/100 µg/ml ampicillin for 18–24 h. Both *cfp* and *yfp* plasmids were extracted from the cultures obtained using a Biomiga plasmid extraction kit following the manufacturer's protocol. The extract yield was checked by electrophoresis. The coding sequences for *cfp* and *yfp* were separately recovered from their respective plasmids by PCR. The 5′ primer was targeted to the N-end of the gene, and included the *Xba*I site. It had the following nucleotide sequence:5′-cgtctagaatggtgagcaagggcgaggagctgttc-3′. The 3′ primer was targeted to the C-end of the gene and contained a *Hind*III site and of the sequernce: 5′-cgaagcttttacttgtacagctcgtccatgccgag-3′. The PCR product was electrophoresed, and the preparations that showed the proper bands were extracted using a gel extraction kit. The extracted preps of the *cfp* and *yfp* genes were separately ligated into the *Xba*I/*Hind*III sites of the recombinant pMALE-*ptsG* plasmid. The ligated reaction mix was transformed into competent *E. coli* DH5α cells and plated onto LB/ampicillin (100 µg/ml) plates. Selected transformants were subjected to plasmid extraction followed by *Xba*I/*Hind*III restriction cutting of the extracted plasmid preps. The extracted plasmids that showed the proper restriction maps for cloned *cfp* and *yfp* were subjected to DNA sequencing using the first primer that annealed near the end of *ptsG* and of the sequence 5′-gcagcgggcgtagtggttg-3′, while the second primer was a reverse primer that annealed downstream the cloned *cfp* or *yfp* gene and was of the sequence 5′-cattcgccattcaggctgcgcaac-3′. The resulting constructs are called pMALE-*ptsG-cfp* and pMALE-*ptsG-yfp*. The N-terminus of the *ptsG* gene was fused to *malE* contained in the pMALE plasmid. Recombinant plasmids pMALE-*ptsG-cfp* and pMALE-*ptsG-yfp* were separately electroporated into *E. coli* strain BL21DE3.

#### Production of the MBP-II^Glc^-CFP/YFP fusion proteins

Fluorescent fusion proteins were generated essentially as described by [8]. The recombinant cells were grown in double strength LB containing 0.2% glucose and induced with 0.5 mM isopropyl β-D thiogalactoside (IPTG) 2 h before harvesting (12–14 h incubation period). The cells were harvested by centrifugation, washed 3x with modified M63 (MM63), pH 7.2, consisting of (g/l): KH_2_PO_4_, 13.3 and (NH_4_)_2_SO_4_, 2 and resuspended in 35 ml of the same medium containing 2 mM dithiothreitol (DTT) and 1 mM ethylenediamine tetraacetic acid (EDTA). The cells were lyzed by passage through a French press 3× at 15,000 psi. The unbroken cells and cell debris were removed by centrifugation at 10,000 RPM for 10 min; then the crude cell extract was centrifuged using a Beckman ultracentrifuge at 40,000 rpm for 2 h in a Titanium 70 rotor. The 2 h HSS produced was gel filtered as described by [1], [8] and the fractions corresponding to activity peaks 1 and 2 were separately collected for purification. Occasionally, peak 2 CFP was obtained using the whole 2 h HSS of the respective strain grown under the same conditions except that after 2 h induction, the culture was treated with 4% paraformaldehyde (pH 10.5) to a final concentration of 0.8% for 30 min, followed by inactivation with 2.5 M glycine (pH 3.2) to a final concentration of 125 mM (5 min with shaking under standard growth conditions). The cells were then harvested, washed with MM63/1 mM DTT/1 mM EDTA and treated as described before, yielding the 2 h HSS.

#### Protein and Enzyme assays

Protein concentrations were determined with the DC Bio-Rad colorimetric protein assay (Cat. #500-0006) using bovine serum albumin as the standard. The transphosphorylation (TP) activity was determined as described by [21]. The substrate was 100 µM [^14^C]methyl-α-glucoside, and the phosphate donor was 10 mM glucose-6-phosphate.

#### *In vitro* FRET analysis of MBP-II^Glc^-CFP/MBP-II^Glc^-YFP mixtures

*Intra- and interactions between peaks 1 and 2.* Protein-protein interactions of II^Glc^ in gel filtration peaks 1 and 2 of the 2 h HSS of *E. coli* BL21DE3(pMAL-*ptsG-cfp*) and BL21DE3(pMAL-*ptsG-yfp*) were studied by FRET analyses. The MBP-II^Glc^-CFP was excited at 436 nm where emission at 488 nm can be absorbed by MBP-II^Glc^-YFP. The excitation of YFP resulted in emission at 526 nm. The purified protein sample in the elution buffer (20 mM Tris•HCl, 200 mM NaCl, 1 mM EDTA, 1 mM DTT, 10 mM maltose) was contained in a 100 µl quartz cuvette. Measurements were carried out in a spectrofluorometer [Fluorolog-3 (FL 3–22), Jobin yvon Inc. (Horiba group)] with a 2.5 or 3.5 slit for both excitation and emission. For detection of FRET and protein-protein interactions, the following samples were examined: (i) CFP fused protein plus YFP fused protein from either peak 1 or peak 2 were mixed after purification, (ii) CFP fused protein plus YFP fused protein from either peak 1 or peak 2 were mixed, purified and then analyzed, (iii) CFP fused protein plus YFP fused protein from peak 1 were mixed, triton treated, purified and then analyzed, (iv) CFP fused protein plus YFP fused protein from peak 1 were separately purified, mixed, triton treated and then analyzed, (v) CFP fused protein from peak 2 plus YFP fused protein from peak 1 were mixed, purified and then analyzed. Detergent treatment was done using triton X100 at a final concentration of 0.1% with stirring or shaking at 4°C overnight. In each case, CFP fused protein alone and YFP fused protein alone from the corresponding peak and in amounts equivalent to that present in the mixture under examination were similarly treated and analyzed. Enzyme purification was conducted by affinity chromatography on an amylose resin as described by [8]. Columns containing 12 ml of amylose resin were washed with 8 column volumes of buffer (20 mM Tris•HCl, 200 mM NaCl, 1 mM EDTA and 1 mM DTT). After sample loading, each column was washed with 12 bed volumes of column buffer and eluted with column buffer containing 10 mM maltose. The pooled fractions were concentrated in an Amicon concentrator, 30,000 MWCO. The eluate was centrifuged using a fixed angle rotor at 6,000 rpm for 25 min. The concentrated samples of mixed or separate peaks were collected, and their protein concentrations were determined. The purified preparations were used for FRET analyses. The samples were excited at 436 nm, and the emission resulting in each case was monitored over the wave length range 440–700 nm at 2 nm intervals. The apparent FRET efficiency was calculated as the ratio of the emission intensities at 526 and 476 nm.

*Verification of protein protein interactions between II^Glc^ molecules of MBP-EII^Glc^-CFP and MBP-EII^Glc^-YFP fused proteins.* This was carried out by testing: (i) the addition of un-labeled II^Glc^-MBP to different peak 1 mixes of MBP-II^Glc^-CFP/MBP-II^Glc^-YFP, (ii) the removal MBP by specific enzymatic digestion with factor Xa.

For (i), validation of the method was conducted by: (a) testing the interference of un-labeled II^Glc^-MBP (its separate effect on the emission of labeled MBP-II^Glc^-CFP and MBP-II^Glc^-YFP), (b) determining the concentration of II^Glc^-MBP which can be used without interference with the emission of reactants. A control experiment without un-labeled II^Glc^-MBP was carried in parallel. Peak 1 of *E. coli* BL21DE3(pMALE-*ptsG-cfp*) was mixed with the Peak 1 fraction of BL21DE3(pMALE-*ptsG-yfp*) in the presence of unlabeled peak 1 of BL21DE3(pMALE-*ptsG*). Un-labeled peak 1 of BL21DE3(pMALE-*ptsG*) was similarly included in the separate test samples of CFP and YFP. The different mixtures produced were solubilized with Triton X100 and purified through an amylose column.

For (ii), digestion with factor Xa was carried out moderately or excessively. The digestion mixture contained 20 mM Tris-HCl, pH 8, 200 mM NaCl, 1 mM EDTA, 1 mM DTT, 4 mM CaCl_2_ and 10 mM maltose. Factor Xa was added at 0.1 or 0.2 µg of enzyme protein per 1 µg of sample protein. The reaction mixture was kept at room temperature (23°C) for 12 and 34 h, then analyzed by 10% SDS gel electrophoresis and FRET measurements. Digestion was also performed at 23°C for 3 days using 0.3 µg of enzyme per 1 µg of sample protein, and the digested samples were subjected to FRET analyses. Excessive digestion with factor Xa was carried out in two stages, the first stage with 0.3 µg of enzyme per 1 µg of sample protein for 3 days, and the second stage at 0.2 µg of enzyme per 1 µg of sample protein for a further 3 days. Digested samples were subjected to SDS PAGE which was carried out essentially as described by [8] and FRET analyses.

*Processing and presentation of the data.* Emission over the wavelength range 440–700 nm for YFP alone was subtracted from that of the CFP plus YFP mixture. Then, the profile produced (termed corrected CFP) was plotted against the profile of CFP alone. In such cases, one can detect the change in the CFP profile in the CFP plus YFP mixture due to the presence of YFP. In positive FRET, one can see a decrease in the peak at 476–488 nm and an increase in emission over the range around 526 nm. The ratio of emission at λ476/emission at λ526 for CFP alone and CFP in the CFP plus YFP mixture after subtraction of YFP (corrected CFP) were determined and compared. In positive FRET, a decrease in the ratio of the emission at λ476/emission at λ526 for the corrected CFP was observed as compared to that of CFP alone.

### *In vivo* cross linking

*E. coli* strain BW25113Δ*ptsG*Δ*malE::km*(pMALE-*ptsG)* (generated in this study, see NMR methodology below) was grown in double strength yeast extract-tryptone broth (2× YT, Difco) containing 0.2% glucose and 100 µg/ml ampicillin at 37°C/275 rpm for 8 h. The cells were induced with IPTG at a final concentration of 0.5 mM for 2 h, and then a 4% paraformaldehyde (PFA) solution was added to the required concentration. Paraformaldehyde (polymer of HCHO, Fisher company, used as a source of formaldehyde) was dissolved in phosphate buffered saline or distilled water to get a 4% stock solution. To achieve solubility, NaOH pellets were added to a final pH of about 10.5. Incubation was continued under the same conditions for 30 min, then PFA action was stopped by the addition of 2.5 M glycine (pH 3.2) to the final concentration of 125 mM, and the culture was left under the same condition for an additional 5–10 minutes. The cells were harvested immediately, washed 3× with MM63/1 mM DTT/1 mM EDTA, and then resuspended in the same buffer. The procedures were completed as described by [1] to get the pellet fraction and the 2 h HSS (French Press: 3× at 16,000 PSI). The following preparations: low speed pellet, high speed pellet and high speed supernatant were obtained and analyzed (as described before) for protein content and transphosphorylation activity. The transphosphorylation (TP) assay was done at 100 µl sample fraction and 100 µl of assay substrate (100 µM [^14^C] methyl-α-glucoside/10 mM glucose-6-phosphate), with a reaction time was 2 h.

### *In vitro* cross linking

The effect of chemical cross linking with formaldehyde on enzyme II^Glc^ activity of peaks 1 and 2 obtained from gel filtration of the 2 h HSS of *E. coli* strain BW25113Δ*ptsG*Δ*malE::km*(pMALE-*ptsG)* was tested. *E. coli* cells were cultured and treated as described under *in vivo* cross linking except that no paraformaldehyde was added. The 2 h HSS obtained was gel filtered as described [1] to yield peaks 1 and 2. A freshly prepared paraformaldehyde stock solution was added to the required concentration, and the mixture was left at 37°C for 30 min under shaking conditions. The formaldehyde reaction was stopped by inactivation with glycine at 125 mM final concentration for 5 min at room temperature (RT).

### Particle size characterization by Cryo-electron microscopy and dynamic light scattering

Particle size characterization of the II^Glc^-MBP preparations was performed by Cryo-electron microscopy (EM) and dynamic light scattering (DLS). The samples treated included purified II^Glc^-MBP of pellets, peak 1 or peak 2 of *E. coli* strain BL21DE3(pMAL-*ptsG*), each at 1–2 mg/ml protein concentration. The samples were dissolved in Tris buffer, pH 7.4, containing 1 mM EDTA and 1 mM DTT.

**Electron microscopic** studies were carried out at the Cryo-electron microscopy facility (UCSD). Uranyl acetate (2% aqueous solution) was used as a negative stain. Negative staining was done on carbon-coated 1000-mesh copper grids that were glow-discharged (in a partially evacuated chamber while applying high voltage where the electron potential ionizes the gas within the chamber, and the negatively charged ions are deposited on the carbon, giving the carbon film an overall hydrophilic surface) before staining. One- to 5-µl samples were adsorbed for up to 1 min, the grid was rinsed with 2–3 large drops of ddH_2_O to remove interfering salts/buffer components, and samples were then stained with freshly filtered 2% uranyl acetate. The sample was placed on an electron microscopy grid and was rapidly frozen at liquid nitrogen temperatures. By maintaining specimens in liquid nitrogen or kept colder, they can be introduced into the high-vacuum of the electron microscope column. The grid was examined on the same day.

**Dynamic light scattering (DLS)** is a technique used for measuring the size of molecules and particles undergoing Brownian motion by observing time-dependent fluctuations in the intensity of scattered light [22]. In DLS, the sample is illuminated with a laser beam, and the intensity of the resulting scattered light produced by the particles fluctuates at a rate that is dependent upon the size of the particles. Analysis of these intensity fluctuations yields the diffusion coefficient of the particles and hence the particle size. This technique is non-invasive and used for characterizing macromolecules in solution and particles in suspension [23], [24], [25].

Measurements were made on a Zetasizer Nano Series instrument (Malvern Instruments LTD) at 25°C, and the viscosity value for water was used in all measurements. The size and percentage of particles (percentage size distribution) were expressed in terms of intensity, volume or number and graphed as peaks. Also, the *z*-average particle size was provided. TheZ-average is the mean particle size obtained from the diffusion coefficient; it averages all the particles in the scattering volume and is therefore sensitive to the presence of large particles [26].

### NMR analysis

#### Bacterial strains

*E. coli* strain BL21DE3 (pMALE-*ptsG*) [8] was used for preparation of different enzyme fractions (pellet, peak1 and peak 2) that were purified using an affinity amylose column and used for NMR studies. In some cases, *E. coli* strain BW25113Δ*ptsG*Δ*malE::km*(pMALE-*ptsG)* (generated in this study) was also used for affinity purification of II^Glc^-MBP from pellet, peak 1, peak 2 and peak 2 of P1-free 2 h HSS obtained from lysates of cells subjected to *in vivo* cross linking. Peak 2 of P1-free 2 h HSS was obtained from cultures of *E. coli* strain BW25113Δ*ptsG*Δ*malE::km*(pMALE-*ptsG)* subjected to *in vivo* cross linking at 0.8% paraformaldehyde (PFA) added after a 2 h induction with IPTG at 0.5 mM (see above for details).

#### Generation of the recombinant *E. coli* strain BW25113Δ*ptsG*Δ*malE::km*(pMALE-*ptsG)*

The recombinant plasmid pMALE-*ptsG* [8] was transferred into the double mutant *E. coli* strain BW25113Δ*ptsG*Δ*malE::km* by electroporation. Generation of the double mutant was carried out principally according to the procedures of [27]. *E. coli* strain BW25113, which harbors a temperature sensitive recombinant plasmid, pKD46, carrying the gene of the recombinase enzyme, was used for generation of the double mutant by homologous recombination. The different primers and plasmids used are listed in Table 1. The kanamycin (*km*) gene was separately introduced into BW25113 to replace the *ptsG* or *malE* gene; then it was removed from the resultant *ptsG* mutant to give the deletion mutant BW25113Δ*ptsG*. The Δ*malE*::*km* mutation was transferred by P1 phage into the *ptsG* deletion mutant.

**Table 1:** Primers and plasmids used in generating the *E. coli* strain BW25113Δ*ptsG*Δ*malE::km* double mutant.

Primer code	Sequence	purpose
PptsG1-P1	5′-ctaacctgcaaaaggtcggtaaatcgctgatgctgccggtatccgtac**tgtgtaggctggagctgcttcg**-3′ target gene vector	Cloning of the *km* gene from plasmid pKD4 to generate the *ptsG* mutation.
PptsG2-P2	5′-tcaggttatcggatttagtaccgaaaatcgcctgaacaccagaaccag**catatgaatatcctccttag-3′** target gene vector	
PmalE-P1	5′-cgatgatgttttccgcctcggctctcgccaaaatcgaaggtaaac**tgtgtaggctggagctgcttcg-3** target gene vector	Cloning of the *km* gene for the *malE* mutation.
pmalE-P2	5′-gtctttcagggcttcatcgacagtctgacgaccgctggcggcgttgat**catatgaatatcctccttag-3** target gene vector	
Kt	5′-cggccacagtcgatgaatcc-3′	Verification of *km.*
K2	5′-cggtgccctgaatgaactgc-3′	
malE-R	5′-gcattacttggtgatacgag-3′	Verification of the *malE* mutation together with K2.
malE-F	5′-cacgagcacttcaccaacaaggac-3′	Verification of *malE* mutation together with either Kt or male-R.
ptsG-F	5′-gcacccatactcaggagcactctc-3′	Verification of the *ptsG* mutation together with Kt.
Plasmid	Purpose	
pKD46	Encodes phage λRed recombinase under an inducible promoter, easily curable (temperature sensitive, low number copy) to assist homologous recombination of the *km* resistance gene.	
pKD20	A helper plasmid encoding the FLP recombinase; also easily curable by growth at 37^o^C (temperature sensitive replicons). It contains an arabinose-inducible P*_ara_BAD* promoter and acts on the directly repeated FRT (FLP recognition target) sites flanking the resistance gene. The transacting II protein is the product of the *pir* gene.	
pKD4	Template plasmid for cloning the *km* gene; contains an FRT-flanked kanamycin resistance (*km*) or chloramphenicol resistance (*cat*) gene.	

#### Enzyme purification for NMR study

The fused II^Glc^-MBP was purified from the different fractions (pellet, peak 1, peak 2 or different fractions of P1) essentially as described by [8]. No detergent was applied to any crude fraction unless specifically stated, and the pellet fraction was homogenized by magnetic stirring at 4°C for 24 h before loading on a column in diluted form (0.1 mg/ml).

#### NMR experiments

The experiments were conducted using Varian Mercury NMR 300 (VNMR hg300). The following conditions were applied: nucleus ^31^P, nucleus solvent, 10% D_2_O; transmittance frequency (Sfrq.), 121.472 MHz, decoupler frequency (dfrq.), 300.078 MHz; decoupler nucleus, H1; power level for decoupler (dpwr), 40; acquisition time (at), 1.165 or 1.598 sec; number of transients (nt), up to 46,000; observed transmittance power (tpwr), 60; pulse width (pw), 15 µs; line broadening (lb), 150; spectral width (sw), 27000; temperature (temp), 25, 37 or 45°C. The ^31^P chemical shift referred to was that of 85% H_3_PO_4_ which was used in a 5-fold diluted form (17%). The NMR tubes applied were of NORELL brand (part: S-5-600-7, 5 mm, 600 MHz), distributed by Cambridge Isotope Laboratories, Inc.

### Phospholipid analyses of different preparations of II^Glc^-MBP

#### Strain used, growth conditions and purification of II^Glc^-MBP

BL21DE3 carrying pMALE-*ptsG* was grown, and the procedures followed were as described above (see FRET analyses methodology) for preparation of pellet, peak 1 and peak 2 fractions and enzyme purification.

#### Lipid extraction

The total polar lipids from purified II^Glc^-MBP of peaks 1 and 2 and the crude pellet fraction of *E. coli* strain BL21DE3(pMALE-*ptsG*) grown under induced conditions were extracted. In parallel, and to be used as controls, total polar lipids of crude pellet fractions of *E. coli* strains BL21DE3(pMALE-*ptsG*) (non induced culture), BL21DE3 and wild type 301 were prepared. Extraction was conducted according to [28], [29] as follows. An aliquot of 0.5 ml of a sample containing 0.25% MgCl_2_ was mixed with 3.75 ml methanol by vortexing. Then, 7.5 ml of CHCl_3_ was added, the mixture was incubated for 1 h at room temperature in a shaker, and then phase separation was induced by adding 2.35 ml of 0.25% aqueous MgCl_2_. The extract was left for 10 min at room temperature and then centrifuged at 1000 RPM for 10 min. The lower (CHCl_3_) phase was collected, and the upper phase was washed twice, each with 5 ml of the pure solvent mixture, the composition of which was equivalent to the assumed composition of the lower phase (CHCl_3_/methanol/water, 86∶14∶1, v/v/v) containing 0.25% MgCl_2_ by shaking for 15 min. Combined organic phases were washed twice, each with 10 ml of the pure solvent mixture, whose composition was equivalent to that of the upper phase (CHCl_3_/methanol/water, 3∶48∶47, v/v/v) containing 0.25% MgCl_2_ by gentle stirring at room temp for 30 min. At each step, flushing with nitrogen gas was performed. Combined organic phases were dried in 1 ml aliquots in a vacuum centrifuge at 35°C and stored at −20°C.

#### Lipid analysis by LC/ESI mass spectrometry

Machines used: LC (Ultrafast Microprotein analyzer, Michron Bioresources, Inc.) and Mass Spectrometry (Finnigan LC QDECA). The following analysis parameters were applied: ESI Source Voltage: −4.5 kV, Sheath Gas Flow: 80.0 PSI, Auxiliary Gas Flow: 20.0 PSI, Capillary Voltage: −15.0 V, Capillary Temperature (C): 275°C.

*Phosphatidylethanolamine (PE) and phosphatidylglycerol (PG) analysis.* LC/Mass spectrometry for PE and PG analysis was performed using a reverse phase column (Clipeus C18, 5 µM, 150X2.1 mm, P/N: Cs-1521-C185, S/N 971553, Ha/Higgins Analytical, Inc., made in USA). Mobile phase A (A) consisted of 50% acetonitrile (ACN)/50% H_2_O/20 mM ammonium formate while mobile phase B (B) consisted of 20% ACN/80% isopropanol/20 mM ammonium formate. The gradient (0.2 ml/min) was as follows: 0–5 min, 0% B; 5–7 min, 0% B to 100% B; 7–45 min, 100% B; 45–46 min, 100% B to 0% B; 46–50 min, 0% B.

For standard (STD), internal standard (IS) and sample preparation, methyl alcohol (MeOH) was used for dissolving the dried materials as well as for dilutions to get the final concentration, and each of the standards and samples was injected in pure MeOH. The injected amount of both PE and PG standards was 50 ng each; PE internal standard, 50 ng; PG internal standard, 5 ng. Between assay runs, the system was flushed with mobile phase B for 10 min followed by mobile phase A for another 5 min. The standards/internal standards mixture was run both at the beginning and at the end of the assay. For sample analysis, both PE (50 ng) and PG (5 ng) internal standards were included in the injected samples. For phospholipids quantitation, Xcalibur 1.3 software from the Thermo Finnigan Corporation was used.

*Cardiolipin (CL) analysis.* Cardiolipin was analyzed according to[30] using normal phase column (Microsorb Si HPLC column, Cat.# 80–125, serial # 10011, Rainin instrument company, Inc.). Mobile phase A consisted of methanol/water, 9∶1 with 0.1 ml per liter of 25% aqueous ammonia. Mobile phase B consisted of CHCl_3_ with 0.1 ml per liter of 25% aqueous ammonia. For sample and standard preparation, the dried lipid was dissolved in a solution consisting of 80% mobile phase B and 20% mobile phase A. The flow rate applied was 800 µl/min. A flow splitter after LC and before mass spectrometer was applied, so 12.5% of the sample (100 out of 800 µl) went to the mass spectrometer. The gradient (0.8 ml/min) was as follows: 0–4 min, 80% B; 4–10 min, 80% B to 50% B; 10–15 min, 50% B to 0% B; 15–17 min, 0% to 80% B; 17–20 min, 80% B. The injected amount of cardiolipin (CL) standard was 300 ng, and CL internal standard was 150 ng. Between assay runs, the system was flushed with mobile phase A for 5 min followed by mobile phase B for another 10 min. The standard/internal standard mixture was run both at the beginning and at the end of the assay. For sample analysis, 150 ng internal standard was included in the injected samples.

For phospholipid quantitation, Xcalibur 1.3 software from the Thermo Finnigan Corporation was used.

## Results

### *In vitro* FRET analyses of MBP-II^Glc^-CFP/MBP-II^Glc^-YFP mixtures

**Figure 1:**
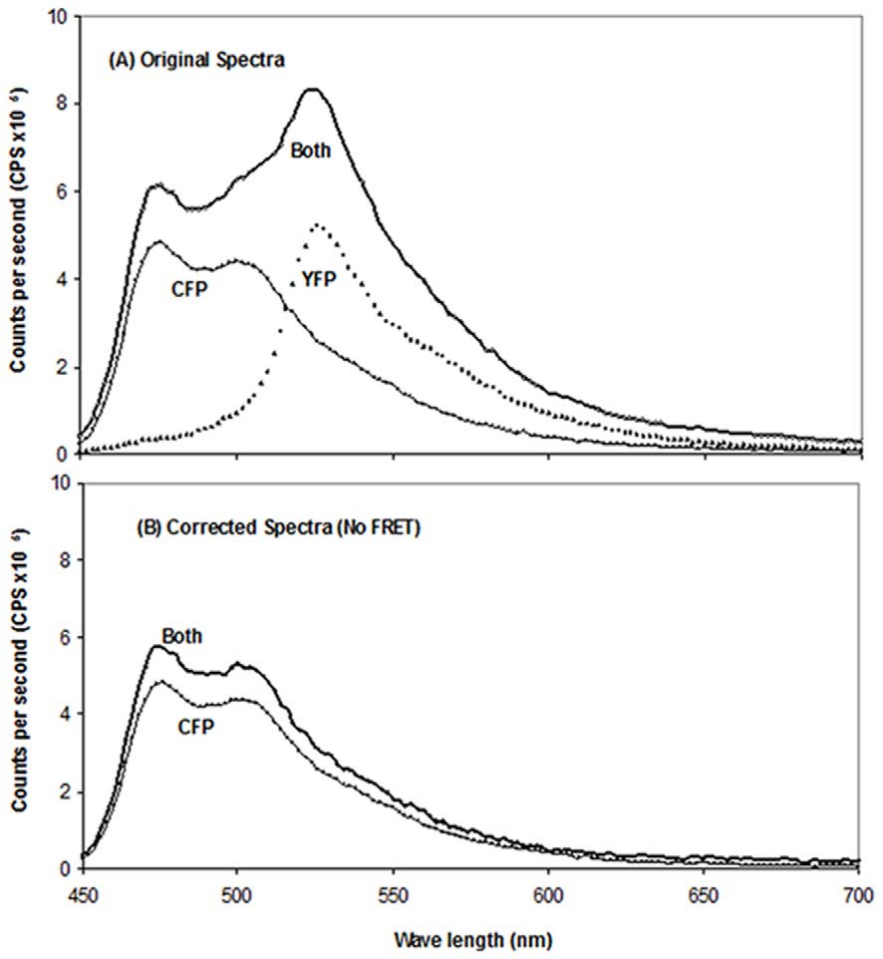
Fluorescence spectra of purified Peak 1 CFP-II^Glc^ plus Peak 1 YFP-II^Glc^ mixture, purified Peak 1 CFP-II^Glc^ and purified Peak 1 YFP-II^Glc^. (A) The two fluorescent proteins were not detergent-treated, but were mixed before purification at a 1:6 ratio (Both; dark line). Alternatively, the spectrum of pure Peak 1 CFP-II^Glc^ (CFP, light line) or pure Peak 1 YFP-II^Glc^ (YFP, dotted line) alone was measured. (B) The spectrum of the YFP-II^Glc^ protein was subtracted from the spectrum of the Peak 1 CFP-II^Glc^ - Peak 1 YFP-II^Glc^ mixture (Both corrected, dark line). The purified CFP-II^Glc^ spectrum is also shown (CFP, light line). (No FRET was observed).

The expressed II^Glc^, fused at its N-terminus to the maltose-binding protein, MBP, and at its C-terminus to either cyan-fluorescent protein, CFP, or yellow-fluorescent protein, YFP, in the 2 h high speed supernatant (HSS), was gel filtered to produce peaks 1 and 2. The fused proteins, either CFP or YFP, were purified separately or after mixing as described under Methods. For each peak, and for both peaks together, fluorescent resonance energy transfer, FRET, analyses of the MBP-II^Glc^-CFP/MBP-II^Glc^-YFP mixtures were carried out. FRET analyses of mixtures of Peak 1 (P1)-CFP and P1-YFP fused proteins, mixed either before or after purification, were carried out at CFP/YFP ratios of 1/1, 1/6, 1/10 and 1/20 (some are presented in Table 2) and the results revealed that in no case was a FRET signal observed. When mixing was done before purification and at a CFP/YFP ratio of 1/6 (total protein concentration of 1.87 mg/ml), the emission profiles of the mixed CFP and YFP fusion proteins as well as their individual counterparts are shown in Figure 1A while the emission profiles of the corrected CFP in the CFP plus YFP fused protein mixture and CFP fused protein alone were as shown in Figure 1B. The emission at λ476/emission at λ526 ratio for the donor (CFP) alone was 1.87. The same ratio for the CFP + YFP mixture after subtracting the contribution due to YFP (corrected CFP) was 1.85. The emission measurements were repeated at various concentrations of total protein and various ratios as mentioned above, but in no case was FRET observed (Table 2). Therefore, there is no evidence that II^Glc^ subunits of the two preparations have undergone shuffling.

**Table 2:** Summary of FRET analyses demonstrating interactions between MBP-II^Glc^-CFP and MBP-II^Glc^-YFP fused proteins of peaks 1 and 2.

Preparation	Mixing condition	CFP/YFP ratio of fused proteins	Protein concentration (mg/ml) of the CFP and YFP fused protein mixtures	emission at λ 476/emission at λ 526 for CFP (control)	emission at λ 476/emission at λ 526 for CFP of corrected CFP (CFP in the CFP and YFP mixture)^a^	FRET result (arbitrary FRET intensities)
Peak 1	Purified CFP and YFP fused proteins were mixed after purification.	1	0.71	1.91	1.85	Nearly no FRET
			0.5	1.9	1.82	
		1/6	4.93	1.92	1.99	
		1/10	4.93	1.93	2.05	
	Crude CFP and YFP fused proteins were mixed, and then purified.	1/6	1.87	1.87	1.85	Nearly no FRET
			0.94	1.79	1.94	
		1/10	1.85	1.88	1.79	
			0.93	1.87	1.77	
	Crude CFP and YFP fused proteins were mixed, detergent treated, purified and then analyzed.	1/6	2.2	1.96	1.24	Presence of FRET (36.7%)
		1/10	2.49	1.96	1.18	Presence of FRET (39.8%)
		1/20	2.15	1.97	1.08	Presence of FRET (45.2%)
	Purified CFP and YFP fused proteins were mixed, detergent treated and analyzed.	1/6	4.69	1.85	1.97	No FRET
		1/10	4.69	1.84	1.94	
Peak 2	Purified CFP and YFP fused proteins were mixed after purification.	1	0.51	1.9	1.77	Nearly no FRET
		1/6	1.64	1.88	1.97	
			4.85	1.93	1.99	
		1/21	0.55	1.87	2.8	
	Crude CFP and YFP fused proteins were mixed and then purified.	1/10	1	1.88	1.22	Presence of FRET (35.1)
		1/16	0.61	1.91	1.46	Presence of FRET (23.6)
Peak1/Peak 2	Crude CFP and YFP fused proteins from peaks 2 & 1, respectively, were mixed and then purified.	1/6	8.4	1.93	1.54	Presence of FRET (20.2)
		1/3	11.7	1.89	1.34	Presence of FRET (29.1)

a The presence of FRET was indicated by a decrease (>10%) in the ratio of emission at λ476/emission at λ 526 of corrected CFP (CFP in the CFP and YFP mixture after subtraction of the contribution due to YFP) as compared to the ratio of emission at λ476/emission at λ 526 for CFP alone (control).

**Figure 2:**
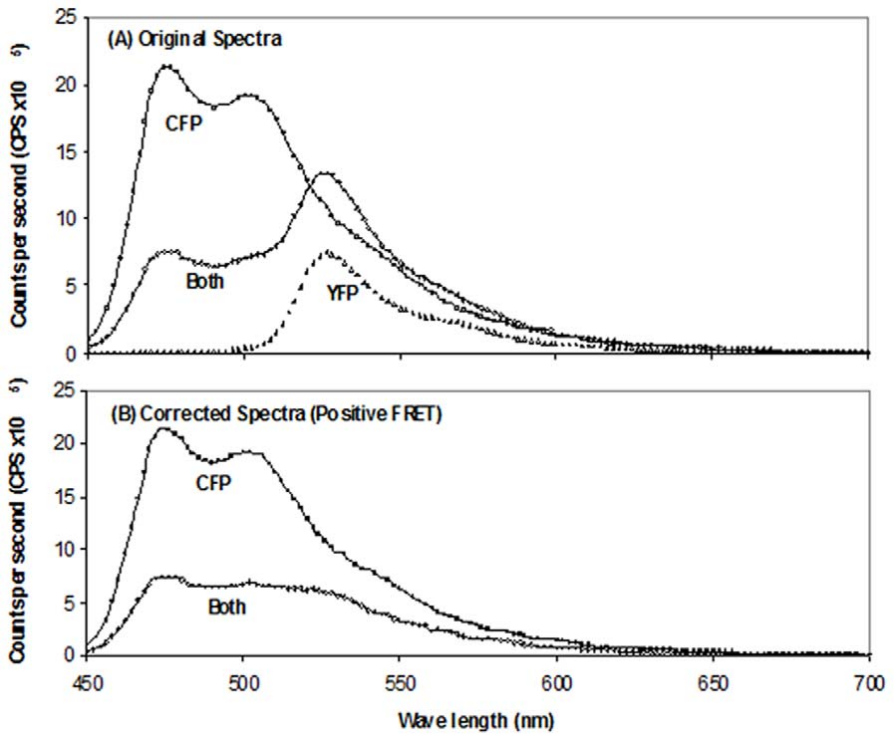
Fluorescence spectra of purified Peak 1 CFP-II^Glc^ plus Peak 1 YFP-II^Glc^ mixture, purified Peak 1 CFP-II^Glc^ and purified Peak 1 YFP-II^Glc^. (A) The two fluorescent proteins were mixed before purification at a 1:6 ratio, detergent treated and then purified (Both, dark line). Alternatively, the spectrum of pure Peak 1 CFP-II^Glc^ (CFP, light line) or Peak 1 YFP-II^Glc^ (YFP, dotted line) alone was treated similarly before the spectrum was measured. (B) The spectrum of the YFP-II^Glc^ protein was subtracted from the spectrum of the Peak 1 CFP-II^Glc^ - Peak 1 YFP-II^Glc^ mixture (Both (corrected), dark line). The purified CFP-II^Glc^ spectrum is also shown (light line). FRET was observed due to detergent treatment preceding purification.

FRET analyses were then performed with mixtures of CFP and YFP fusion proteins of peak 1, mixed, detergent-treated and purified before analyses (CFP/YFP ratios as before). At a CFP/YFP ratio of 1/6, the peak 1 CFP fused protein was mixed with the peak 1 YFP fused protein and treated with Triton X100 as described under Methods. The mixture was purified (amylose resin), and subjected to FRET analyses. The total protein concentration of the purified preparation of the mixed CFP and YFP fused proteins was 2.2 mg/ml. The emission profiles of the individual and mixed CFP and YFP fusion proteins are shown in Figure 2A while the emission profiles of the corrected CFP fusion protein for the mixture and for the CFP fusion protein alone are presented in Figure 2B. The emission at λ476/emission at λ526 ratio for the donor (CFP) was 1.96, while that of the corrected CFP was 1.24. The presence of FRET indicates the formation of hetero II^Glc^-YFP/II^Glc^-CFP dimers.

**Figure 3:**
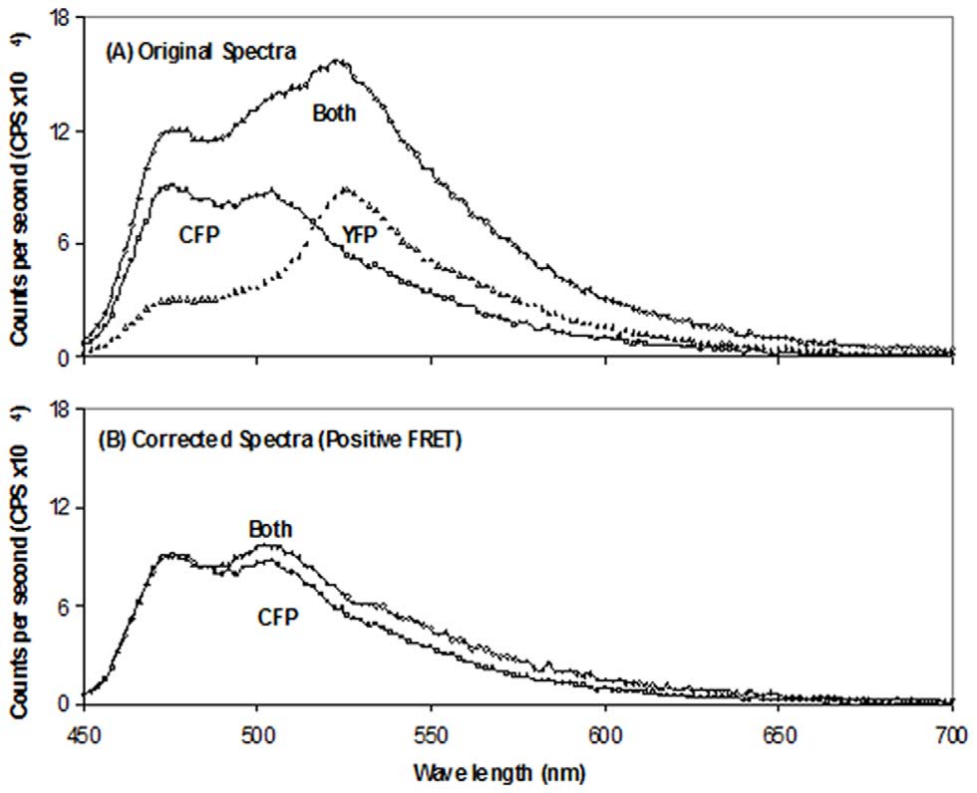
Fluorescence spectra of Peak 2 CFP-II^Glc^ (hereafter designated “CFP”) plus Peak 2 YFP-II^Glc^ (hereafter designated “YFP”). (A) The two fluorescent proteins were mixed at a ratio of 1∶10 before purification (dark line). The light and dotted lines show the spectra for purified Peak 2 CFP and Peak 2 YFP, respectively as indicated. (B) Corrected fluorescence spectra of Peak 2 CFP plus Peak 2 YFP (mixed at a ratio of 1:10 before purification) (both, dark line) and purified Peak 2 CFP alone (light line). FRET was observed in the absence of detergent treatment.

The results in the case of peak 2 are shown in Table 2, and regardless of the protein concentrations and ratios used, FRET was always observed if the mixture was purified on the amylose column after but not before mixing. For example, in the case of peak 2, with a CFP/YFP ratio of 1/10, and where the mixture was subjected to amylose affinity purification, the emission profiles of the mixed CFP and YFP fused proteins as well as their individual counterparts are shown in Figure 3A while the emission profiles of the corrected CFP fused protein in the CFP and YFP fused protein mixture and the CFP fusion protein alone are shown in Figure 3B. The emission ratio at wavelengths 476 and 526 nm for the donor (CFP) was 1.88 and for the corrected CFP of the mixture, it was 1.22, showing that FRET was observed.

**Figure 4:**
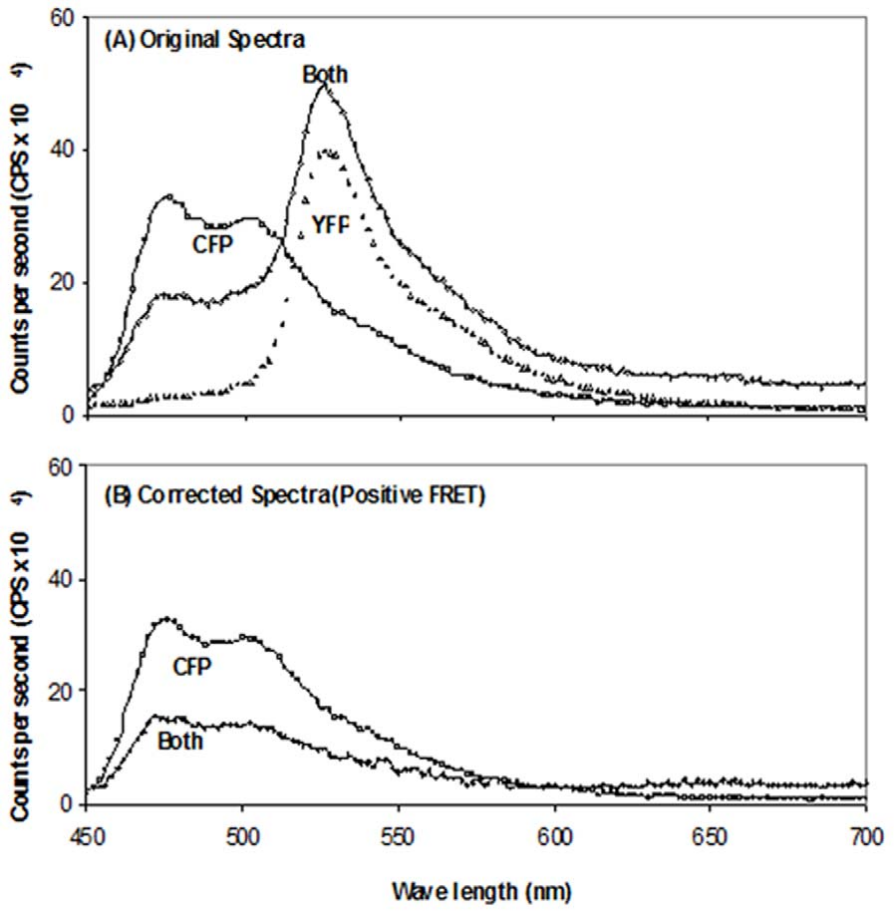
Fluorescence spectra of Peak 2 CFP plus Peak 1 YFP. (A) The two fluorescent proteins were mixed at a ratio of 1∶6 before purification (dark line), and purified Peak 2 CFP and Peak 1 YFP were examined alone as indicated (light and dotted lines, respectively). (B) Fluorescence spectra of corrected Peak 2 CFP plus Peak 1 YFP (Both corrected, dark line) and Peak 2 CFP alone (CFP, light line).

For peak 2/peak 1, with a CFP/YFP ratio of 1/6 and with the mixture subjected to amylose affinity purification, the emission profiles of the mixed CFP and YFP fused proteins as well as their individual counterparts are shown in Figure 4A while the emission profiles for the corrected CFP fused protein in the CFP and YFP fused protein mixture and the CFP fusion protein alone were shown in Figure 4B. The emission ratio at wavelengths 476 and 526 nm for the donor (CFP) was 1.93 and for the corrected CFP was 1.54, showing that FRET was observed. These and other results using the same approach are summarized in Table 2.

As reported previously [31] the inner membrane proteins (IMPs) in Gram negative bacteria like *E. coli* become inserted into the membrane with the aid of certain mediator proteins. YidC has been identified as a factor that assists in the integration, folding, and assembly of IMPs, both in association with the Sec-translocon and separately. Our results suggest that this can also occur *in vitro* between the cytoplasmic form of II^Glc^ and the bilayer dimeric membranous form of II^Glc^. In this regard [32] reported that the prokaryotic signal recognition particle receptor (SR) consists of FtsY and strikingly, although FtsY requires membrane contact for functionality, cell fractionation studies have localized FtsY predominantly to the cytosolic fraction of *E. coli* cells. It has been suggested that the prokaryotic SR might bind the signal recognition particle-SRP-ribosome-nascent chain complex (SRP-RNCs) already in the cytosol, and only then does it initiate membrane targeting. In their report [33], showed that membrane protein targeting in bacteria is mediated by the signal recognition particle (SRP), FtsY and the bacterial SRP receptor (SR). They found that 30% of FtsY in *E. coli* are found stably associated with the cytoplasmic membrane. This could explain in part our observation of the interaction of the II^Glc^ monomeric form with the membraneous form containing the FtsY mediator. However, this interaction may be a spontaneous, unassisted integration process. Further evidence for protein-protein interactions between II^Glc^ monomers was obtained.

#### Testing the addition of un-labeled II^Glc^-MBP to different mixtures of MBP-II^Glc^-CFP/MBP-II^Glc^-YFP

Table 3 shows FRET analyses of interactions between MBP-II^Glc^-CFP and MBP-II^Glc^-YFP fused proteins of peak 1 in the presence of different concentrations of un-labeled II^Glc^-MBP of the same peak. It is obvious that incorporation of un-labeled MBP-II^Glc^ at low concentration had no appreciable effect on FRET signal detection (experiments b & c) while increasing its amount to 32.3 mg abolished the FRET signal without causing interference with the emissions of CFP and YFP alone (exp. d & e). Under the conditions of these two experiments, the ratio of emission at λ 476/emission at λ 526 for CFP alone ranged around 1.9, and that for YFP alone ranged around 0.03. Higher amounts of added un-labeled MBP-II^Glc^ (≥73.5 mg) caused interference in the emission of CFP alone (the ratio of emission at λ 476/emission at λ 526 for CFP ranged between 1.77−1.43) and for YFP alone (the ratio of emission at λ 476/emission at λ 526 for YFP ranged between 0.06–0.8) with no FRET signal (exp. f, g & h). Accordingly, this interference invalidated these three experiments, and the ratio of emission at λ 476/emission at λ 526 of corrected CFP (CFP in the CFP and YFP mixture) showed fluctuating values.

**Table 3:** Summary of FRET analyses of interactions between MBP-II^Glc^-CFP and MBP-II^Glc^-YFP fused proteins of peak 1 in the presence of different concentrations of un-labeled II^Glc^-MBP.

Experiment	Protein amount of crude MBP-II^Glc^-CFP (mg)	Protein amount of crude MBP-II^Glc^-YFP (mg)	Protein amount of crude un-labeled MBP-II^Glc^. (mg)	Ratio of emission at λ 476/emission at λ 526 for CFP	Ratio of emission at λ 476/emission at λ 526 for YFP	Ratio of emission at λ 476/emission at λ 526 for corrected CFP (CFP in the CFP and YFP mixture)^a^	-Interference of added un-labeled II^Glc^-MBP with the emission of reactants-FRET result
Exp. a(control)	5.2	26	0.0	1.94	0.032	1.43	-Positive FRET
Exp. b	5.2	26	9.5	1.87	0.034	1.39	-No interference,-Positive FRET
Exp. c	5.2	26	19.1	1.95	0.026	1.48	-No interference,-Positive FRET
Exp. d	5.2	26	32.3	1.93	0.036	2.38	-No interference,-No FRET
Exp. e	5.2	26	64	1.97	0.0378	2.95	-No interference,-No FRET
Exp. f	5.2	26	73.5	1.78	0.062	2.005	-Little interference,-No FRET,
Exp. g	5.2	26	96.5	1.77	0.087	1.94	-Little interference,-No FRET
Exp. h	5.2	26	338.1	1.43	0.084	2.24	-Interference of added un-labeled II^Glc^-MBP with the emission of reactants.

a The presence of FRET was indicated by a decrease (>10%) in the ratio of emission at λ 476/emission at λ 526 for the corrected CFP (CFP in the CFP and YFP mixture after subtraction of the contribution due to YFP) as compared to the ratio of emission at λ 476/emission at λ 526 for CFP alone (control).

Verification was achieved by examination of specific enzymatic removal of MBP on the positive FRET signal of the MBP-II^Glc^-CFP/MBP-II^Glc^-YFP interacting species. Moderate digestion with factor Xa had nearly no effect on the positive FRET signal of the interacting CFP/YFP species (Table 4). However, extended digestion abolished such positive signals.

**Table 4:** FRET analyses of positive FRET samples of MBP-II^Glc^-CFP/YFP of peak 1 after digestion with factor Xa.

Experiment	Digestion condition	Ratio of emission at λ 476/emission at λ 526 for CFP	Ratio of emission at λ 476/emission at λ 526 for corrected CFP (CFP in the CFP and YFP mixture)^a^	FRET result
Control I	No digestion	1.94	1.17	Positive FRET
factor Xa- digested sample (I)	Digestion was carried out at 0.3 µg of enzyme protein per 1 µg of sample protein for 3 days (Moderate digestion).	2	1.38	Positive FRET
Control II	No digestion	1.9	1.14	Positive FRET
factor Xa- digested sample (II)	Digestion was carried out in two stages, the first stage at 0.3 µg of enzyme protein per 1 µg of sample protein for 3 days followed by a second digestion at 0.2 µg of enzyme protein per 1 µg of sample protein for a further 3 days (Excessive digestion).	1.97	1.97	No FRET

a The presence of FRET was indicated by a decrease (>10%) in the ratio of emission at λ 476/emission at λ 526 for the corrected CFP (CFP in the CFP and YFP mixture after subtraction of the contribution due to YFP) as compared to the ratio of emission at λ 476/emission at λ 526 for CFP alone (control).

**Figure 5:**
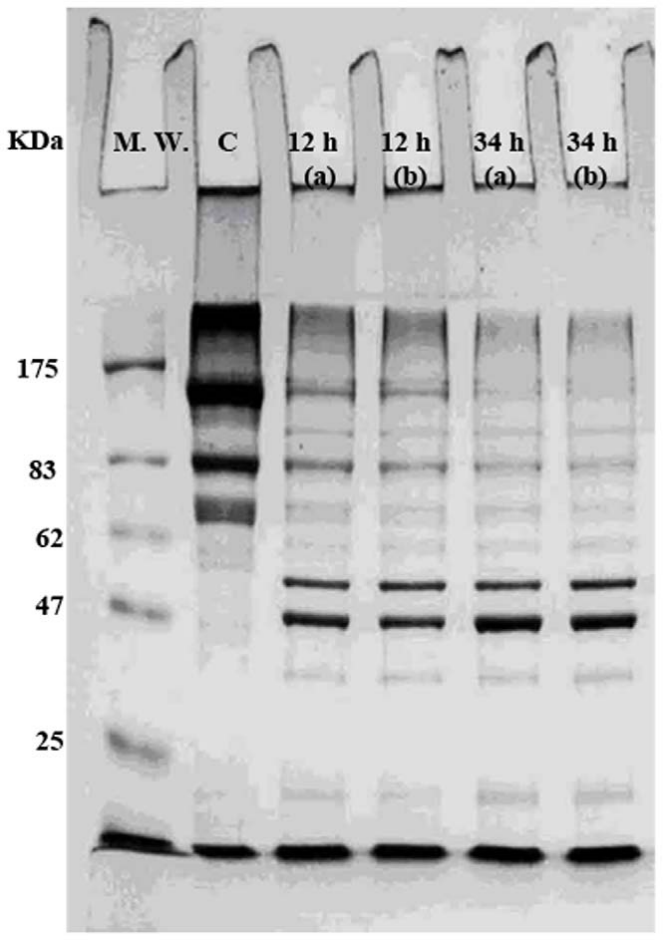
SDS PAGE of factor Xa digested samples of MBP-II^Glc^-CFP/MBP-II^Glc^-YFP. M. W., molecular weight markers; C, factor Xa undigested sample; 12 h a & b, samples were digested for 12 h with 0.1 and 0.2 µg enzyme/µg sample protein, respectively; 34 h a & b, samples were digested for 34 h with 0.1 or 0.2 µg enzyme/µg of sample protein, respectively.

**Figure 6:**
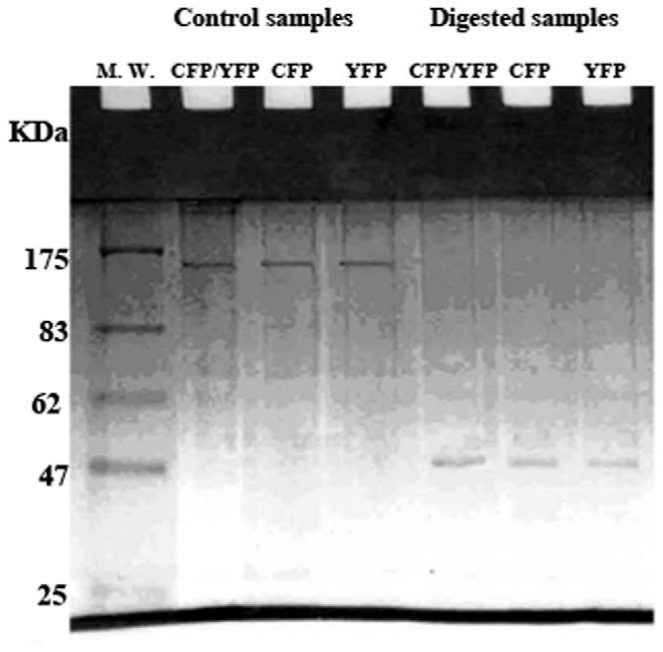
SDS PAGE of FRET positive samples digested with factor Xa of MBP-II^Glc^-CFP/MBP-II^Glc^-YFP. Samples were applied at 1 µg protein per lane. Digestion was carried out in two stages, the first stage at 0.3 µg of enzyme protein per µg of sample protein for 3 days, and the second stage at 0.2 µg of enzyme protein per µg of sample protein for a further 3 days.

The results show that moderate digestion with factor Xa (Figure 5) which affects release of a digested product with a molecular weight of about 40 KDa, comparable to that of MBP, did not affect the FRET signal, excluding the possible involvement of fused MPB in the positive FRET signal of interacting species. However, excessive digestion with factor Xa caused non specific digestion and protein degradation accompanied by the disappearance of the parent proteins, leaving a band with a molecular weight of about 40 KDa, comparable to that of MBP (Figure 6).

### Formaldehyde cross-linking studies of MBP-II^Glc^ preparations, conducted both *in vivo* and *in vitro*

#### *in vivo* cross linking studies

**Figure 7:**
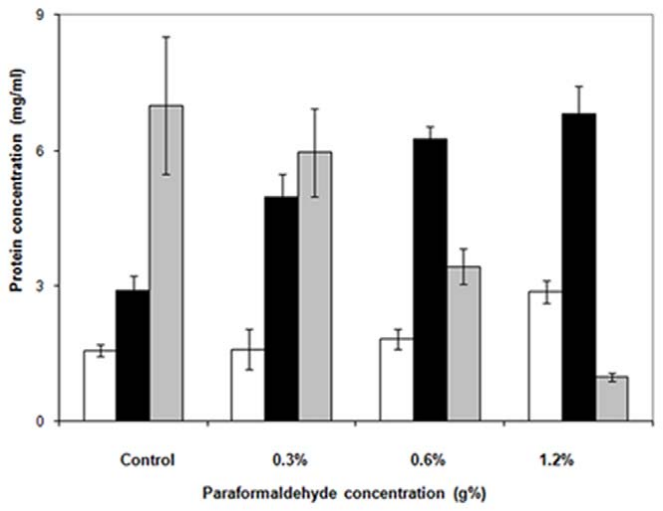
Effect of *in vivo* cross linking with different concentrations of paraformaldehyde (PFA) on the protein concentration of different preparations obtained from the cell lysate of *E. coli* strain BW25113Δ*ptsG*Δ*malE::km*(pMALE-*ptsG)*. Low speed pellets, white bars; high speed pellets, black bars; high speed supernatants, grey bars. Thin lines above the histograms represent the standard deviations (S.D).

**Figure 8:**
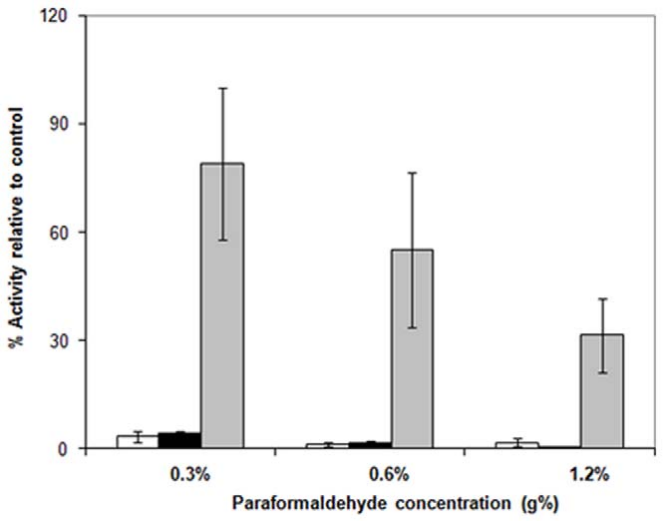
Effect of *in vivo* cross linking with different concentrations of paraformaldehyde (PFA) on transphosphorylation (TP) activity of different preparations obtained from the cell lysate of *E. coli* strain BW25113Δ*ptsG*Δ*malE::km*(pMALE-*ptsG)*. Low speed pellets, white bars; high speed pellets, black bars; high speed supernatants, grey bars. Thin lines above the histograms show the error bar (S.D). Values are expressed in percentage of original samples.

*In vivo* cross linking caused a change in the relative distribution of cell lysate components (low speed pellets, high speed pellets and high speed supernatant) (Figure 7). A decrease in the protein contents of these high speed supernatants was demonstrated by *in vivo* cross linking, and this decrease was proportional to the PFA concentration applied. On the other hand, an increase in the protein content was demonstrated in low and high speed pellets. Accordingly, PFA treatment of growing cells caused agglomeration of soluble proteins to give sedimentable proteins in both low and high speed centrifugation. Concerning enzymatic activity of II^Glc^, the transphosphorylation activities of the three cell lysate fractions (low speed pellets, high speed pellets and high speed supernatant) (Figure 8) showed a dramatic reduction in the activities of low and high speed pellets, even at low PFA concentrations, while the high speed supernatant showed a lower reduction in activity which was proportional to the PFA concentration. This differential reduction in activity is attributed to the differential sensitivity of II^Glc^ of the pellet fractions and that of the high speed supernatant. However, the lower II^Glc^ activity of the high speed supernatant could be attributed to a comparable decrease in the protein contents of the HSS and/or to the action of PFA on II^Glc^ activity *per se*.

**Figure 9:**
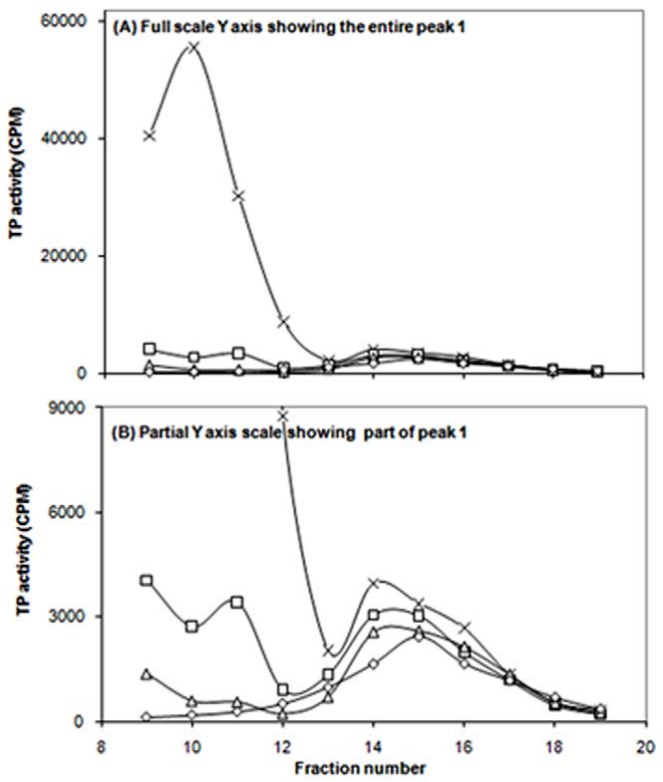
Transphosphorylation (TP) activities of gel filtration fractions of 2 h high speed supernatants prepared from cultures of *E. coli* strain BW25113Δ*ptsG*Δ*malE::km*(pMALE-*ptsG)* that were subjected to *in vivo* cross linking with paraformaldehyde (PFA) at 0.3, 0.6 and 1.2%. Control, x's; 0.3% PFA, squares; 0.6% PFA, triangles; 1.2% PFA, diamonds.

**Figure 10:**
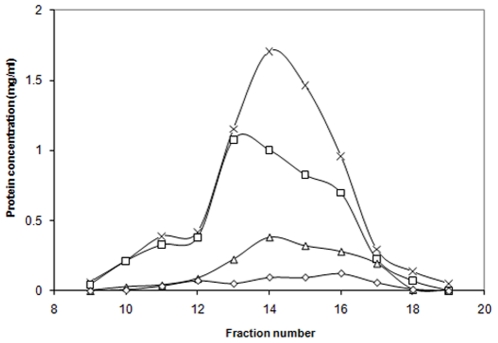
Protein profile of gel filtration fractions of a 2 h high speed supernatant prepared from a culture of *E. coli* strain BW25113Δ*ptsG*Δ*malE::km*(pMALE-*ptsG)* that were subjected to *in vivo* cross linking with paraformaldehyde (PFA) at 0.3, 0.6 and 1.2%. Control, x's; 0.3% PFA, squares; 0.6% PFA, triangles; 1.2% PFA, diamonds.

Figure 9 shows TP activity profiles of gel filtration fractions of 2 h high speed supernatants prepared from cultures of *E. coli* strain BW25113Δ*ptsG*Δ*malE::km*(pMAL-*ptsG*) that were subjected to *in vivo* cross linking with paraformaldehyde (PFA) at 0.3, 0.6 and 1.2%. *In vivo* treatment with PFA affected peak 1 activity much more than peak 2 activity. Regarding the protein profile (Figure 10), decreases in the protein content of the 2 h HSS (represented by peaks 1 and 2) using PFA treatment were proportional to the amount of PFA applied.

#### *In vitro* cross linking

**Figure 11:**
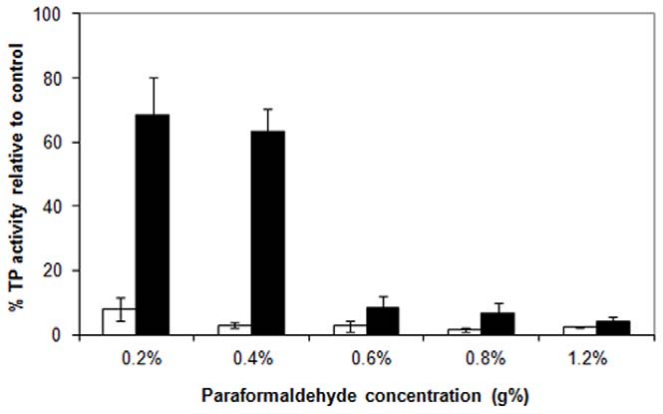
Effect of *in vitro* cross linking with different concentrations of paraformaldehyde on transphosphorylation activity of II^Glc^ peaks 1 (white bars) and 2 (black bars) from *E. coli* BW25113Δ*ptsG*Δ*malE::km*(pMALE-*ptsG)*. The 2 h HSS of a crude cell lysate was gel filtered to get the two activity peaks 1 and 2. Both peaks were subjected to *in vitro* cross linking as described in the Methods section. The thin vertical lines above the histograms represent error bars (S.D).

*In vitro* treatment with PFA of peaks 1 and 2 of *E. coli* BW25113Δ*ptsG*Δ*malE::km*(pMAL-*ptsG*) (Figure 11) showed again (see *in vivo* results for comparison) that peak 1 II^Glc^ was more sensitive to PFA treatment compared than peak 2. Lower concentrations of PFA (0.2 & 0.4%) caused a sharp reduction in peak 1 TP activity while only a 30 to 35% reduction in peak 2 activity was observed. Higher PFA concentrations (0.6 to 1.2%) dramatically affected the TP activities of both peaks (Figure 11).

### Relative sizes of MBP-II^Glc^ preparations determine by cryo-electron microscopy and dynamic light scattering (DLS)

**Figure 12:**
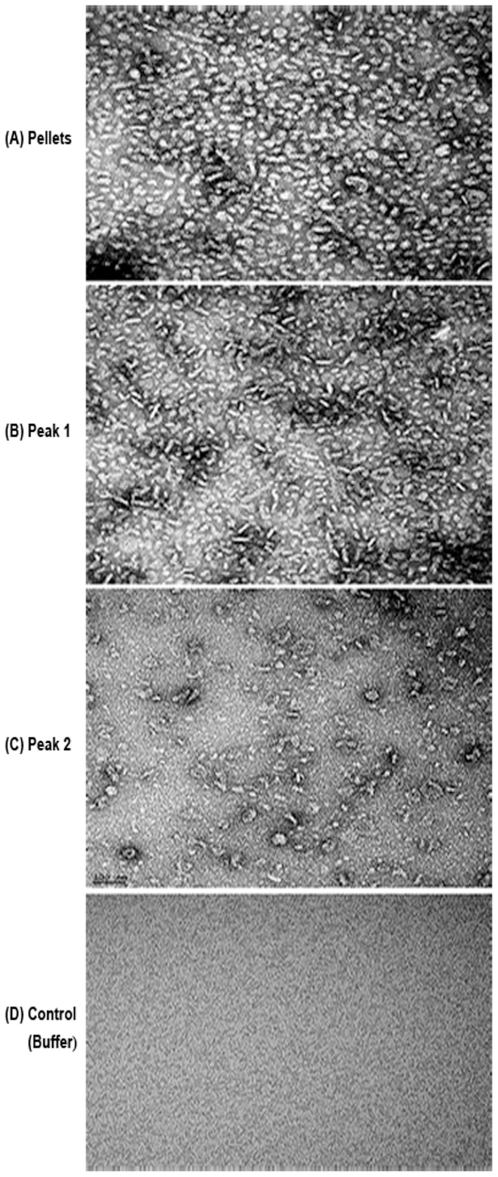
Cryo-electron micrographs of purified II^Glc^-MBP preparations. *E. coli* strain BL21DE3(pMALE-*ptsG)* was used (A) pellet, (B) peak 1, (C) peak 2, (D) buffer control.

Figure 12 shows cryo-electron micrographs of MBP-II^Glc^ preparations obtained from pellet (A), Peak 1 (B) and Peak 2 (C). The figure shows images of different appearances, all at the same level of magnification. The pellets (A) revealed mostly spherical, but also elongated structures, some of the former of which showed central cavities indicative of vesicles. Peak 1 (B) revealed heterogeneity with some spherical and many more elongated particles. Thus, in contrast to Figure 12 (A), the particles are predominantly elongated rather than spherical. In addition, smaller particles not seen in Figure 12 (A) are present in large numbers. The Peak 2 preparation (C) also shows heterogeneity, but small particles predominate while only a few elongated or spherical particles can be seen. These last mentioned structures may have arisen during preparation (see Methods).

**Figure 13:**
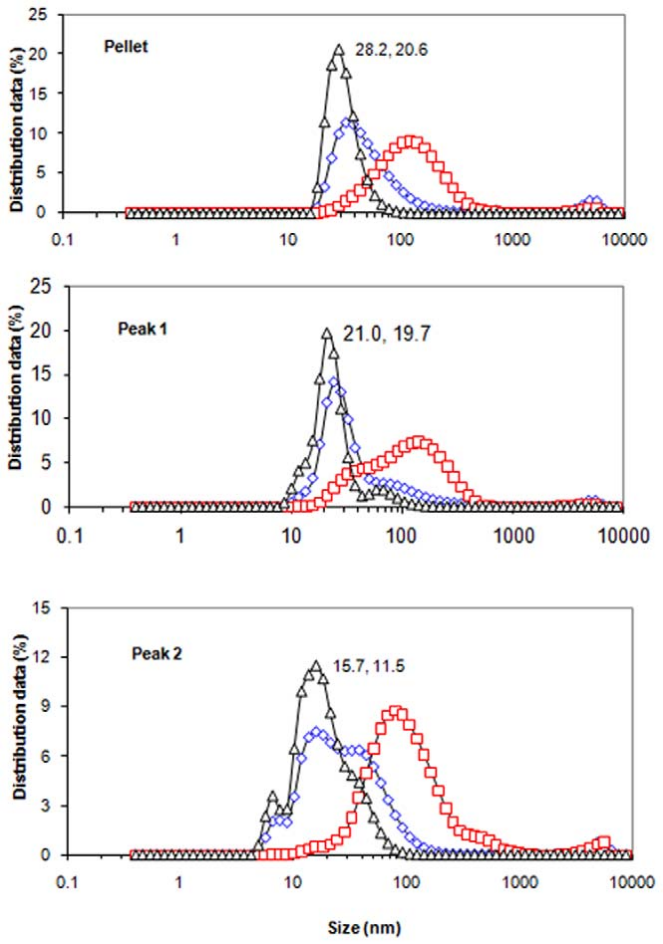
Percentage distribution of II^Glc^-MBP molecular sizes of pellet (top), peak 1 (middle) and peak 2 (bottom) preparations of *E. coli* strain BL21DE3(pMALE-*ptsG)* as determined by DLS. Values are expressed in volume (diamonds), intensity (squares) and number (triangles).

Dynamic light scattering [17], [18] was also used to estimate relative sizes and distributions of particles within these three preparations. The results were obtained in terms of number, intensity and volume (see Figure 13), and the Z average values were also calculated [17], [18]. Estimating the size distributions using number, the value for the pellet peaked at 20.6 nm, that for Peak 1 was at 19.7 nm, and that for Peak 2 was at 11.5 nm. Heterogeneity was apparent from the relative distributions of particle sizes (28.2%, 21.0% and 15.7% for the three preparations, respectively). Z average values for the three preparations were 103 nm, 96 nm and 78 nm for pellet, Peak 1 and Peak 2, respectively. These and other relevant values are summarized in Table 5. The results presented in this section provide relative size distributions of particles within the three preparations, qualitatively in accordance with expectation.

**Table 5:** Summary of DLS analyses.

Preparation	Z-average	Size distribution in terms of number	
	(nm)	size (nm)	Distribution (%)
Pellet	103.1±1.3	28.2	20.5±2.0
Peak 1	96.0±1.0	21.0	19.7±5.4
Peak 2	78.2±6.4	15.7	11.5±2.2

### NMR analyses correlated with enzyme activity measurements

**Figure 14:**
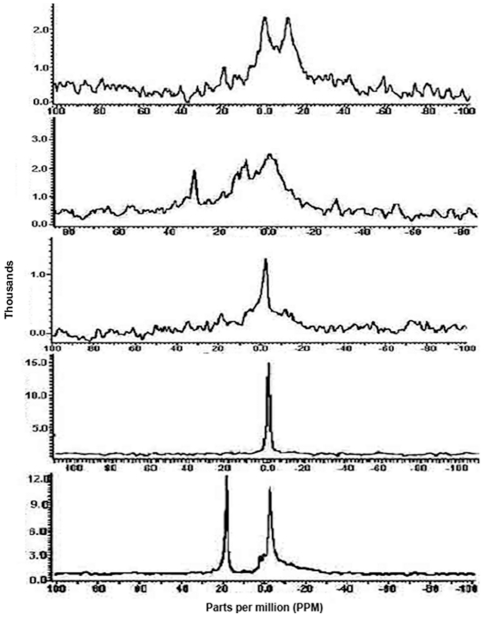
[^31^P]NMR spectra of purified II^Glc^ preparations of pellet (Pt), initial, middle and tail parts of peak 1 (P1-A, P1-B and P1-C, respectively) and peak 2 (P2) of *E. coli* strain BW25113Δ*ptsG*Δ*malE::km*(pMALE-*ptsG)*. The spectra were recorded at 37°C, 46,000 scans and at an acquisition time of 1.6 seconds. Peak 2 was prepared from cells subjected to *in vivo* cross linking. For details see Materials and Methods. The spectra of these tested preparations when examined at 25, 37 and 45°C and at different scans were shown in Figs. S1 to S5 (Saier's web site).

Figure 14 shows the results of ^31^P-NMR spectroscopic analyses of five different preparations of the MBP-II^Glc^ fusion protein when scanned at 37°C for 46,000 scans. The first fraction is the pellet, obtained by high speed centrifugation followed by affinity chromatography (see Methods). The remaining four fractions were obtained by passage of the high speed supernatant (HSS) through a Biogel 1.5 column, separating Peaks 1 and 2 by size exclusion chromatography as reported previously[8]. Peak 1 was subdivided into leading edge, central part, and trailing edge of this peak, where approximately equal volumes of the three fractions comprising Peak 1 were used. These were then re-purified by affinity chromatography and analyzed both by ^31^P-NMR and by enzyme assays. The latter were aimed at quantitating substrate (glucose-6-P) inhibition, a measure of dimerization since only the dimer exhibits this phenomenon [34]. Figure 14 (Pt) shows the NMR spectrum of the phospholipids in the pellet fraction. A typical spectrum normally observed for a bilayer was obtained; it was skewed with an upfield chemical shift anisotrophy. The same profile was obtained, skewed for the leading edge of Peak 1 (Figure 14 (P1-A)), and skewed with a high intensity peak (Figure 14 (P1-B)). By contrast, the trailing edge of Peak 1 as well as Peak 2 showed typical isotropic spectra with no chemical shift, characteristic of micelles (Figures 14, P1-C and P2). The central part of Peak 1 (Figure 14, P1-B) showed intermediate behavior, suggestive of a mixed population or of an intermediate species. These results provide convincing evidence that (1) lipids in the pellet are essentially in bilayer form, as is true of the leading edge of Peak 1, (2) lipids in the trailing edge of Peak 1 as well as Peak 2 are in micellar form, and (3) the central part of Peak 1 exhibits an intermediate composition. Nearly the same behavior was observed when the different preparations mentioned before were analyzed at 25 and 45°C (Figures. S1, S2, S3, S4 and S5 at http://www.biology.ucsd.edu/~msaier/supmat/micellarPTS).

**Figure 15:**
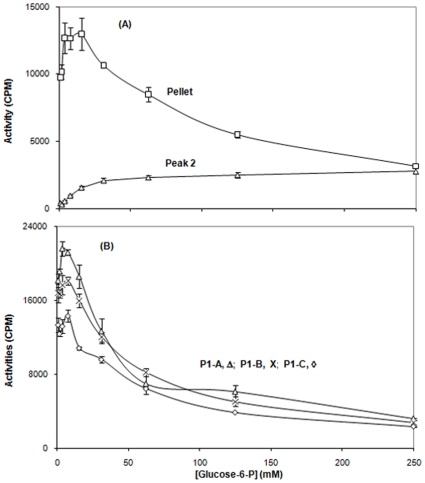
Transphosphorylation (TP) activities of pellet (Top; A; squares) and Peak 2 (Top; A; triangles), as well as Peak 1 (A (leading edge; triangles), B (central part; x's) and C (trailing edge; diamonds)) fractions (Bottom; B). Purified II^Glc^-MBP was prepared from *E. coli* strain BW25113Δ*ptsG*Δ*malE::km*(pMALE-*ptsG).* The radioactive substrate was 100 µM [^14^C]methyl α-glucodside, used with different concentrations of glucose-6-phosphate as indicated.

These five fractions were assayed for [^14^C]-methyl α-glucoside phosphorylation activity as a function of substrate (Glucose-6-P) concentration. Surprisingly, pellet and all three Peak 1 fractions showed essentially the same behavior (Figure 15 (A & B)). They exhibited high activity at relatively low glucose-6-P concentrations, but strong substrate inhibition at high glucose-6-P concentrations [3], [4], [5], [34], [35]. Only peak 2 exhibited a lack of stubstrate inhibition, and high activity was observed only at high sugar-phosphate concentrations (Figure 15 (A)). Because substrate inhibition in the transphosphorylation reaction is characteristic of the dimeric enzyme and not the monomeric enzyme [1], [2], [3], [35], we suggest that while peak 2 is a micellar monomer, the trailing edge of Peak 1 contains a micellar dimer (see Discussion).

### Polar phospholipid analyses of II^Glc^-containing preparations

**Figure 16:**
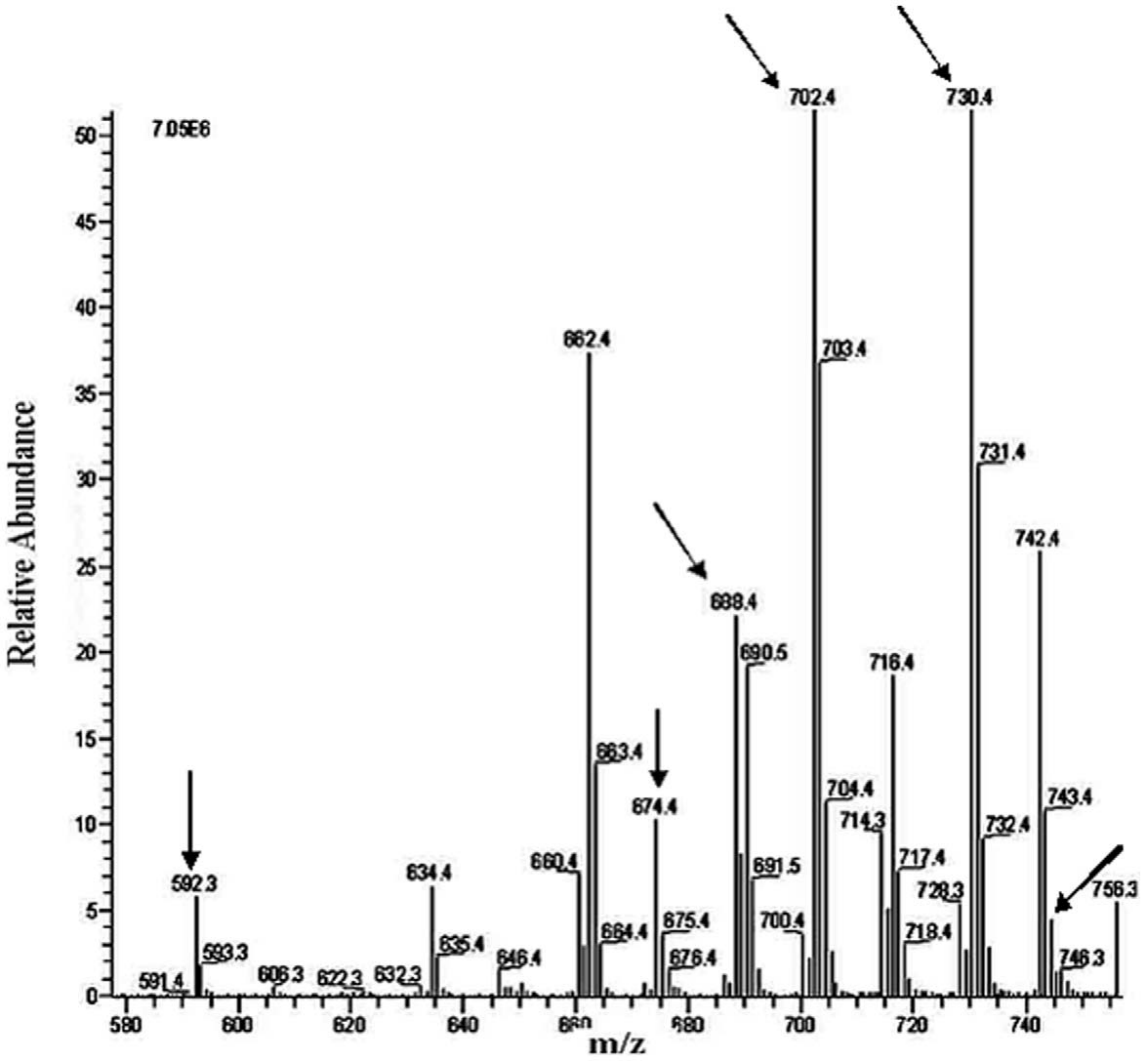
LC-ESI-MS negative ion mode spectra of phosphatidylethanolamine (PE) molecular species of lipid extract of peak 1 fraction of *E. coli* strain BL21DE3(pMALE-*ptsG)*. Arrows indicate different PE molecular species detected in peak 1 and standard preparations (for standard preparation see Figure S6 at Saier web site). I.S., internal standard; right handed numbers at the top of the Y axis indicate full-scale intensity in arbitrary units. The lipid contents of *E. coli* strain BL21DE3(pMALE-*ptsG)* were extracted and analyzed as described in Materials and Methods.

**Figure 17:**
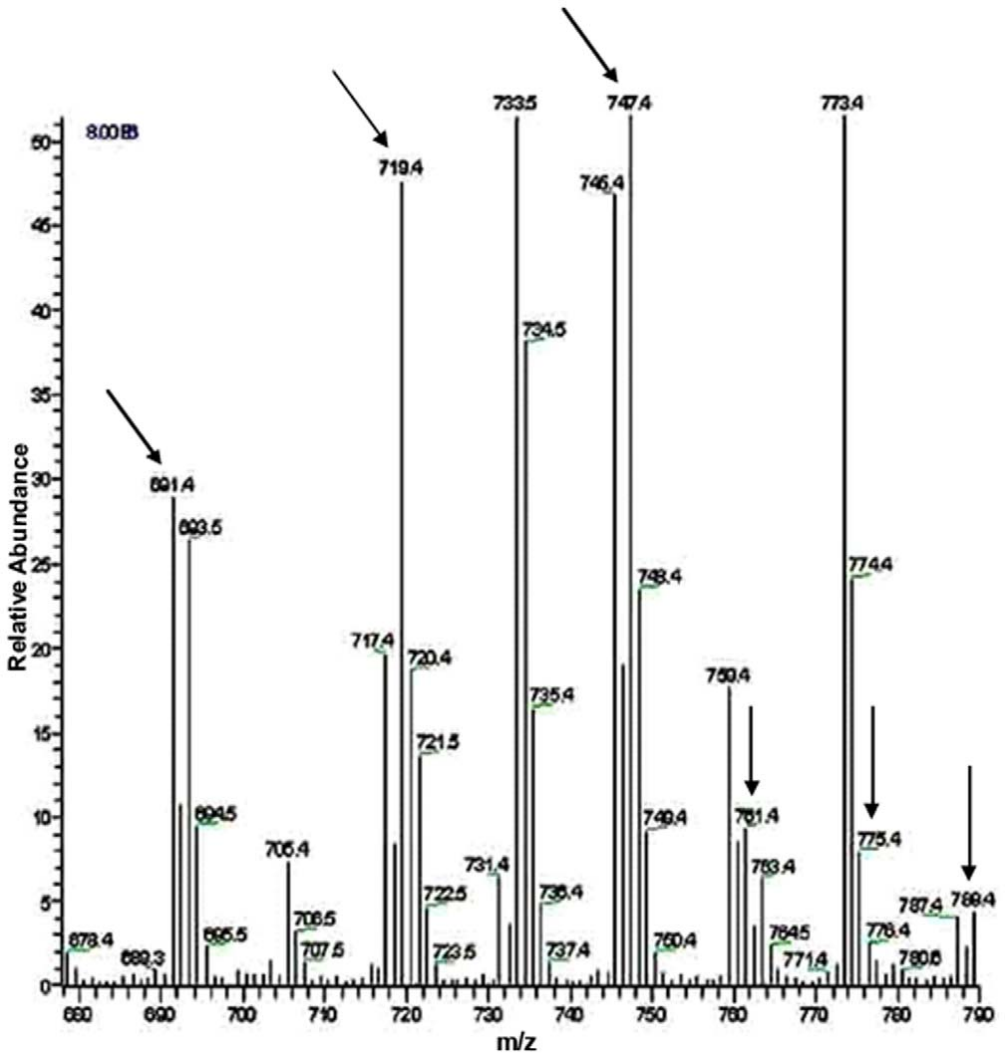
LC-ESI-MS negative ion mode spectra of phosphatidylglycerol (PG) molecular species of lipid extract of peak 1 fraction of *E. coli* strain BL21DE3(pMALE-*ptsG)*. Arrows indicate different PG molecular species detected in peak 1 and standard preparations (for standard preparation see Figure S9 at Saier web site). Right handed numbers at the top of the Y axis indicate full-scale intensity in arbitrary units. The lipid contents of *E. coli* strain BL21DE3(pMALE-*ptsG)* were extracted and analyzed as described in Materials and Methods.

**Figure 18:**
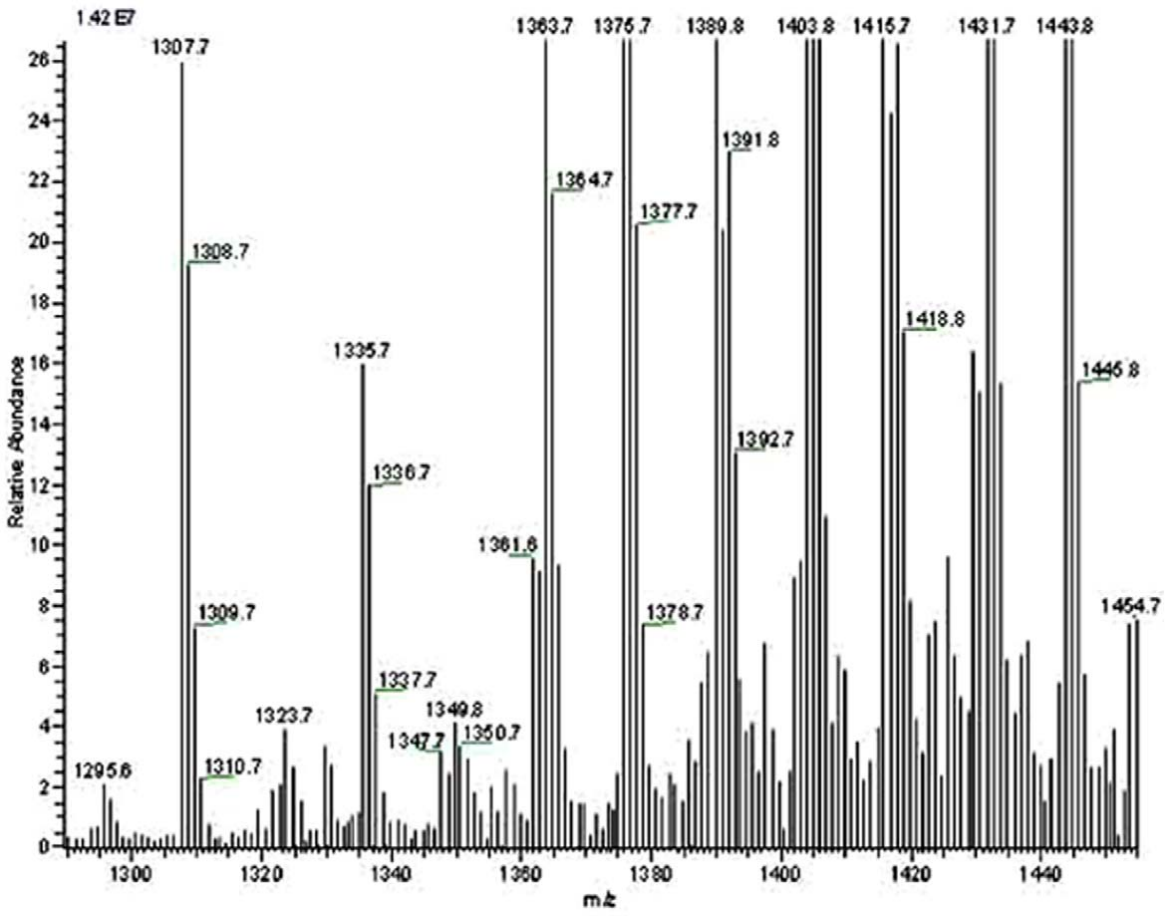
LC-ESI-MS negative ion mode spectra of cardiolipin (CA) molecular species of lipid extract of peak 1 fraction of *E. coli* strain BL21DE3(pMALE-*ptsG)*. Arrows indicate different CA molecular species detected in peak 1 and standard preparations (for standard preparation see Figure S12 at Saier web site). I.S., internal standard; right handed numbers at the top of the Y axis indicate full-scale intensity in arbitrary units. The lipid contents of *E. coli* strain BL21DE3(pMALE-*ptsG)* were extracted and analyzed as described in Materials and Methods.

The polar lipids were extracted from the various preparations (a) whole *E. coli* membrane pellets (2 hr at 40,000 rpm, titanium rotor; ∼200,000×g); (b) the Biogel 1.5 column Peak 1 (gel filtration), and (c) Biogel 1.5 column Peak 2. Each of these was passed through an affinity amylose column (N.E. Biolabs # E8021L) before being subjected to lipid extraction (see Methods). The results obtained are presented in Table 6 while figures 16, 17, 18 show results of the three major *E. coli* phospholipids [PE (Figure 16), PG (Figure 17) and CA (Figure 18)] for peak 1, analyzed by liquid chromatography-mass spectrometry (LC-MS) while the corresponding results for the standard, pellet and peak 2 preparations are shown in Figs. S6, S7, S8 (PE), S9 to S11 (PG) and S12 to S14 (CA) at http://www.biology.ucsd.edu/~msaier/supmat/micellalPTS. The results clearly indicate that the phospholipids in the membranes (pellet) occur in the proportions of PE:PG:CL 61.8∶21∶17.2 which approximates the expected proportions reported for *E. coli* [36], [37]. The values for both P1 and P2 indicate that both of these fractions have decreased ratios of zwitterionic (PE) phospholipid to anionic (PG + CL) phospholipids. Surprisingly, these values were similar for Peaks 1 and 2.

**Table 6:** Relative ratios of PE, PG and CA and lipid/protein ratios of different EII^Glc^-MBP preparations.

Preparation	%PE	%PG	%CA	PE/PG	PE/CA	PG/CA	PE/(PG+CA)	Lipid/protein (ng/ug)
Peak 2 - *E. coli* BL21DE3(pMALE-*ptsG*) (induced prep)	51.3	28.5	20.1	1.8	2.5	1.4	1.0	5.3
Peak 1 *E. coli* BL21DE3(pMALE-*ptsG*) (induced prep)	51.0	30.7	18.3	1.7	2.8	1.7	1.0	11.7
Pellet *E. coli* BL21DE3(pMALE-*ptsG*) (induced prep)	61.8	21.0	17.2	2.9	3.6	1.2	1.6	32.3
Pellet *E. coli* BL21DE3(pMALE-*ptsG*) (non-induced prep)	65.5	23.9	10.6	2.7	6.1	2.2	1.9	25.3
Pellet *E. coli* strain 301 (wild type)	64.5	22.5	12.9	2.8	5.0	1.7	1.8	46.8
Average Pellet	64.0	22.5	13.6	2.8	4.9	1.7	1.8	34.8

The ratios of lipid to protein were also calculated (see Table 6) showing that membranes possess the highest lipid: protein ratios while Peak 2 has the lowest lipid:protein ratio (pellet:P1:P2  = 34.8∶11.7∶5.3). These results, taken together, imply that the different preparations have differing phospholipid compositions as well as different amounts of lipid per unit protein. While Peaks 1 and 2 have similar lipid ratios, the lipid to protein ratio of peak 2 is only half that of P1.

## Discussion

In earlier publications we described the novel finding that each of several integral membrane transport proteins of the bacterial phosphotransferase system (PTS) can exist in a soluble form. Evidence was presented that these soluble Enzyme II complexes were present in the cytoplasm of intact cells [1], [2]. This last mentioned observation has been confirmed in the present study, and we use a variety of physical methods to characterize the various forms of the glucose-specific Enzyme II complex (II^Glc^). The approaches used in this study included:

1. fluorescent energy transfer (FRET),
2. formaldehyde cross linking, both *in vivo* and *in vitro,*
3. measurements of enzyme activity and substrate inhibition,
4. cryo-electron microscopy (cryo-EM) and dynamic light scattering (DLS) for particle size determination,
5. nuclear magnetic resonance (NMR),
6. measurements of relative amounts of the three principal *E. coli* phospholipids, and
7. measurements of the phospholipid to protein ratios.

A brief summary of the results obtained with these seven approaches is presented in Table 7.

**Table 7:** Approaches used in this study and results obtained.

Technique		Nature of the information obtained
1	Fluorescence energy transfer (FRET)	Protein:protein interactions. Peak 2 can associate to peak 1 without detergent. Peak 1 cannot dissociate to peak 2 without detergent.
2	Formaldehyde cross linking	Protein:protein interactions. *In vivo*: peak 1 becomes insoluble with formaldehyde but peak 2 does not. *In vitro*: peak 1 is inactivated; peak 2 is not.
3	Enzyme activity	Dependency of substrate inhibition on monomeric versus dimeric form: identification of dimeric micellar II^Glc^.
4	Cryo-electron microscopy (C-EM) and dynamic light scattering (DLS)	Purified French pressed membrane vesicles are heterogeneous; sizes: pellet> peak 1> peak 2
5	Nuclear magnetic resonance (NMR)	Pellet is only in bilayer form. Peak 1, leading edge: only in bilayer form; peak 1 central part: mixture of bilayer form and dimeric micelles; peak 1 trailing edge: dimeric micelles. Peak 2 monomeric micelles.
6	Lipid measurements	Pellet: PE/PG = 1.8Peak 1: PE/PG = 1.0Peak 2: PE/PG = 1.0
7	Lipid/protein measurements	Pellet: lipid/protein (ng/µg) = 35Peak 1: lipid/protein (ng/µg) = 12Peak 2: lipid/protein (ng/µg) = 5

### Protein-protein interactions of II^Glc^ molecules in peaks 1 and 2, based on FRET analyses

FRET analyses of mixtures of P1 CFP and P1 YFP fused proteins, mixed before or after purification, showed no appreciable FRET signals at the different tested protein concentrations and ratios, suggesting the absence of intermolecular exchange between the II^Glc^ proteins. However, when these same preparations were exposed to Triton X100 before purification, FRET emission spectra revealed excited state energy transfer. This indicates that the II^Glc^-CFP and II^Glc^-YFP fused proteins were in direct contact. Thus, detergent treatment of the stable dimeric mixed Peak 1 CFP and YFP fusion proteins followed by purification on an amylose resin must have caused dissociation to monomers and re-association to give mixed dimers. These results attest to the presence of stable dimers in Peak 1 II^Glc^ unless exposed to detergent.

FRET analyses of mixtures of P2 CFP and P2 YFP fusion proteins, mixed before or after purification and carried out at different ratios of CFP/YFP in the total absence of detergent yielded different results. When purified individually and mixed, no FRET signal was observed, but when mixed and then purified, FRET signals were observed. The most reasonable interpretation is that the monomeric II^Glc^ of peak 2 is stable, but exposure to the amylose resin causes these monomers to associate to dimers. This result confirms earlier observations as reported in [1], [2]. The interpretation of this result will be presented below.

### Formaldehyde crosslinking, electron microscopy, dynamic light scattering and II^Glc^ activity measurements

Utilizing four very different techniques, formaldehyde crosslinking, II^Glc^ activity measurements, cryo-electron microscopy and dynamic light scattering (DLS) in combination, it was possible to gain evidence for several novel features of the different forms of II^Glc^.

First, as expected from the centrifugation and gel filtration results reported previously [1], [2] it became clear from the cryo-electron microscopy and DLS studies that the three fractions, pellet, P1 and P2, showed very different properties. The pellet consists mostly of large tubular and vesicular structures characteristic of French Press disrupted *E. coli* membranes [38]. The enzyme must be in a dimeric form (as indicated by the strong substrate inhibition) in which it is very sensitive to formaldehyde inactivation. P1 proved to also contain a mixture of membranous vesicles and tubules, but more numerous smaller structures were also detected. Based on the NMR results (see below), it appears that many of these medium sized particles are present as dimeric micelles. These putative dimeric micelles predominate in the trailing edge of P1 while the larger membraneous particles (presumably membrane vesicles) were found largely in the leading edge of P1. P2 consists primarily of smaller particles, corresponding to the expected size of lipid containing monomeric micelles. These conclusions were substantiated by the substrate inhibition analyses that showed that pellet and P1 show strong and similar degrees of substrate inhibition while P2 showed none. Previous studies have established that only the dimeric species of a PTS Enzyme II complex exhibits this phenomenon [3], [4], [39].

### Nuclear Magnetic Resonance (NMR) Data

Studies using ^31^P-NMR have proved of great value in understanding the problems of lipid polymorphism in biological membranes [40] and ^31^P-NMR is a powerful tool that can be used to distinguish lipid bilayers from lipid micelles. When hydrated phospholipid samples are subjected to ^31^P- NMR analysis under conditions of proton decoupling (a process which removes the dipolar interactions between protons and phosphorus nuclei) a variety of spectral lineshapes may be encountered. These spectra are sensitive indicators of the physical phase adopted by the lipids. The NMR results showed that as expected, pellet and P2 were present almost exclusively as bilayers and micelles, respectively. Surprisingly, however, P1 also contains substantial amounts of micelles especially in the trailing edge. This result was not expected, because the Enzyme assays had shown that P1-II^Glc^ exhibited substrate inhibition (characteristic of the dimer), comparable to that of the pellet. This means that the micelles present in P1 were most likely dimeric. This report therefore provides the first evidence for dimeric micelles present in P1 but not in appreciable amounts in the pellet or P2.

Occasionally, the isotropic peak 2 spectrum shows an additional spectral component. Such a component was also observed by [41] for micellar spectra and were interpreted as representing a tubular lipid phase. Comparing the results obtained here with lipid analysis data, it can be mentioned that the micellar structure observed for P2 could be correlated with high anionic lipid (PG and CL) content in peak 2 material relative to its PE content. This suggests that a high (PG+CL)/PE ratio favors and stabilizes micellar structure over bilayer structure. Such a conclusion is supported by the following studies.

Kleinschmidt and Tamm [41] used solid-state ^31^P-NMR spectroscopy to determine the minimal chain lengths of some phospholipids that are required for the formation of lipid bilayers. They found that when the chemical shift anisotropies (CSAs) of lipids of the same chain length with different headgroups were compared, an increase of the CSA (i.e. shift to the anisotropic spectra of bilayers) occurred in the order, PG < PE < PC (phosphatidyl choline) < PS (phosphatidylserine). With the exception of PE, this order correlated with the increasing volumes of the lipid headgroups in this sequence. Accordingly, one would generally expect to observe more order (larger CSA) for larger headgroups. These investigators also noticed that the chain-length threshold for bilayer formation follows a different order of phospholipid classes, PE < PC < PS < PG. They reported that in addition to headgroup volume, headgroup charge appears to play an important role in explaining the micelle-to-bilayer transition. Charge repulsion drives negatively charged PGs to the micellar phase and thereby moves this lipid species to the right end of the sequence. The primary amine-containing headgroups of PE and PS can form hydrogen bonds with the phosphate groups of neighboring lipids as demonstrated by crystallography [42], infrared spectroscopy [43], and fluorescence spectroscopy [44], [45]. Hydrogen bonds can also be formed between the hydroxyl groups and the phosphate groups of phosphatidylglycerol molecules [46], but the negative charge remains effective as a repulsive force between PG molecules. The hydrogen-bonding interactions in PE and PS reduce the effective headgroup area and thereby stabilize the bilayer over the micellar structure.

### Lipid and Protein Analyses

All fractions were measured for total lipid and total protein contents. The pellet fractions had the highest lipid to protein ratios while P2 had the lowest such ratio. This fact correlated with our earlier observation that phospholipids stimulate II^Glc^ activity in P2 much more than P1 or pellet II^Glc^ activity [1], [2], [7]. These observations taken together suggest that a lipid deficiency characterizes the monomeric micelles.

The micellar species proved to be enriched in anionic lipids, PG and CL, and deficient for the zwitterionic lipid, PE. This can be explained because II^Glc^ (and other PTS Enzymes II) require a proper balance of PE versus PG+CL for optimal activity [2], [21]. Thus, these enzymes exhibit depressed activity when either the anionic or the zwitterionic lipids are lowered from their natural amounts. The lower lipid to protein ratio in P2 may also contribute to the reduced activity of this preparation.

The physiological impact of micellar species being enriched in anionic lipids, PG and CL, may be of significance. Matsumoto [47] reported that anionic phospholipids recruit peripheral membrane proteins to the membrane by virtue of their negative charges. For example, *E. coli* peripheral membrane proteins such as SecA, FtsY, DnaA, PssA and HtrA (DegP) bind to negatively charged surfaces. In the case of SecA, FtsY, DnaA and PssA, their respective activities are dependent on their binding to major anionic phospholipids. Accordingly, binding of FtsY to the micellar II^Glc^ form might facilitate its insertion in the membrane bilayer. This may be supported by the observation that precursor proteins such as prePhoE and proOmpA were transported (SecA-mediated translocation) across a PG-depleted membrane vesicle when supplemented with CL, PA, phosphatidylserine (PS), phosphatidylethanol or phosphatidylinositol (PI), essentially to the same extent as PG-supplemented membranes [48], [49]. The FtsY protein, an *E. coli* homologue of the alpha-subunit of eukaryotic signal recognition particle receptor, is suggested as having a direct membrane targeting function in protein translocation. *In vitro*, FtsY protein associates efficiently with membrane vesicles containing 30% anionic PG [50]. As in the case of SecA, the membrane association of FtsY is accompanied by a conformational change required for membrane association. After the proteins are recruited to the anionic surface of the membrane, they are inserted into the acyl chain region of the phospholipid bilayer [50], [51], [52]. These proteins are intimately associated with, and surrounded by, hydrophobic acyl chains of phospholipids.

### Overview

The application of physical techniques (FRET, cryo-EM, DLS and NMR) together with standard biochemical approaches (formaldehyde crosslinking, enzyme activity measurements and phospholipid/protein determinations) allow us to come to novel conclusions and hypotheses regarding the nature of the various physical forms of II^Glc^.

1. II^Glc^ can exist in 3 distinct forms: a dimer in a bilayer, a dimer in a micelle, and a monomer in a smaller micelle.
2. Exchange of subunits within the dimer depends on the presence of detergent (Triton X100), but coalescing of monomeric micelles to form dimeric micelles can occur in the absence of detergent.
3. The dimer, previously shown to be required for sugar-P substrate inhibition, is also much more sensitive to inactivation by formaldehyde treatments.
4. Formaldehyde inactivation appears to occur with equal efficiency when the dimer is present in a bilayer or micelle.
5. Particle size decreases in the expected order, pellet>P1>P2, but all preparations are heterogeneous, probably representing in part the ease of particle type interconversion.
6. Nuclear Magnetic Resonance is an ideal method for distinguishing bilayers from micelles, and together with substrate inhibition results, its use allowed us to provide evidence for a previously unsuspected micellar dimeric II^Glc^.
7. The different fractions have different phospholipid:protein ratios (pellet>P1>P2) and different phospholipid types, with the PE contents being pellet>P1>P2.

These advances put the structural features of monomeric and dimeric II^Glc^ micelles on a firm basis and allow us to derive new questions that are subject to experimental examination. The reasons why (1) the dimeric species are more susceptible to formaldehyde inhibition than the monomeric forms, (2) why the monomeric micellar II^Glc^ has more anionic phospholipids than the dimeric species, and (3) what the basis for lipid depletion in the monomeric micellar species have yet to be determined. Future studies will address these questions.

## Supporting Information

Figure S1 **[^31^P]NMR spectra of purified II^Glc^ preparations of pellet (Pt) fraction of** ***E. coli*** **strain BW25113Δ** ***ptsG*** **Δ** ***malE_Kn_*** **-pMAL-** ***ptsG*** **.** The spectra were recorded at different temperatures (25, 37, 45°C), an acquisition time of 1.6 seconds and 46,000 scans. The sample was exposed sequentially to the applied temperatures. For details see Materials and Methods.
(TIF)

Figure S2 **[^31^P]NMR spectra of purified II^Glc^ preparation of initial (P1-A) fraction of peak 1 of** ***E. coli*** **strain BW25113Δ** ***ptsG*** **Δ** ***malE_Kn_*** **-pMAL-** ***ptsG*** **.** The spectra were recorded at different temperatures (25, 37, 45°C), an acquisition time of 1.6 seconds and 46,000 scans. The sample was exposed sequentially to the applied temperatures. For details see Materials and Methods.
(TIF)

Figure S3 **[^31^P]NMR spectra of purified II^Glc^ preparation of middle (P1-B) fraction of peak 1 of** ***E. coli*** **strain BW25113Δ** ***ptsG*** **Δ** ***malE_Kn_*** **-pMAL-** ***ptsG*** **.** The spectra were recorded at different temperatures (25, 37, 45°C), an acquisition time of 1.6 seconds and 46,000 scans. The sample was exposed sequentially to the applied temperatures. For details see Materials and Methods.
(TIF)

Figure S4 **[^31^P]NMR spectra of purified II^Glc^ preparation of tail (P1-C) fraction of peak 1 of** ***E. coli*** **strain BW25113Δ** ***ptsG*** **Δ** ***malE_Kn_*** **-pMAL-** ***ptsG*** **.** The spectra were recorded at different temperatures (25, 37, 45°C), an acquisition time of 1.6 seconds and 46,000 scans. The sample was exposed sequentially to the applied temperatures. For details see Materials and Methods.
(TIF)

Figure S5 **[^31^P]NMR spectra of purified II^Glc^ preparation of peak 2 of** ***E. coli*** **strain BW25113Δ** ***ptsG*** **Δ** ***malE_Kn_*** **-pMAL-** ***ptsG*** **.** The spectra were recorded at different temperatures (25, 37, 45°C), an acquisition time of 1.6 seconds and 46,000 scans. The sample was exposed sequentially to the applied temperatures. For details see Materials and Methods.
(TIF)

Figure S6 **LC-ESI-MS negative ion mode spectra of phosphatidylethanolamine (PE) molecular species in standard preparation.** Arrows indicate different PE molecular species detected. I.S., internal standard; right handed numbers at the top of the Y axis indicate full-scale intensity in arbitrary units. The analysis was done as described in Materials and Methods.
(TIF)

Figure S7 **LC-ESI-MS negative ion mode spectra of phosphatidylethanolamine (PE) molecular species of lipid extract of pellet fraction of** ***E. coli*** **strain BL21DE3-pMAL** ***ptsG*** **.** Arrows indicate different PE molecular species detected in pellet and standard preparations (for standard preparation see Figure S6 at Saier web site). I.S., internal standard; right handed numbers at the top of the Y axis indicate full-scale intensity in arbitrary units. The lipid contents of *E. coli* strain BL21DE3-pMAL*ptsG* were extracted and analyzed as described in Materials and Methods.
(TIF)

Figure S8 **LC-ESI-MS negative ion mode spectra of phosphatidylethanolamine (PE) molecular species of lipid extract of peak 2 fraction of** ***E. coli*** **strain BL21DE3-pMAL** ***ptsG*** **.** Arrows indicate different PE molecular species detected in peak 2 and standard preparations (for standard preparation see Figure S6 at Saier web site). I.S., internal standard; right handed numbers at the top of the Y axis indicate full-scale intensity in arbitrary units. The lipid contents of *E. coli* strain BL21DE3-pMAL*ptsG* were extracted and analyzed as described in Materials and Methods.
(TIF)

Figure S9 **LC-ESI-MS negative ion mode spectra of phosphatidylglycerol (PG) molecular species of standard preparation.** Arrows indicate different PG molecular species detected. I.S., internal standard; right handed numbers at the top of the Y axis indicate full-scale intensity in arbitrary units. The analysis was carried out as described in Materials and Methods.
(TIF)

Figure S10 **LC-ESI-MS negative ion mode spectra of phosphatidylglycerol (PG) molecular species of lipid extract of pellets fraction of** ***E. coli*** **strain BL21DE3-pMAL** ***ptsG*** **.** Arrows indicate different PG molecular species detected in pellet and standard preparations (for standard preparation see Figure S9 at Saier web site). Right handed numbers at the top of the Y axis indicate full-scale intensity in arbitrary units. The lipid contents of *E. coli* strain BL21DE3-pMAL*ptsG* were extracted and analyzed as described in Materials and Methods.
(TIF)

Figure S11 **LC-ESI-MS negative ion mode spectra of phosphatidylglycerol (PG) molecular species of lipid extract of peak 2 fraction of** ***E. coli*** **strain BL21DE3-pMAL** ***ptsG*** **.** Arrows indicate different PG molecular species detected in peak 2 and standard preparations (for standard preparation see Figure S9 at Saier web site). Right handed numbers at the top of the Y axis indicate full-scale intensity in arbitrary units. The lipid contents of *E. coli* strain BL21DE3-pMAL*ptsG* were extracted and analyzed as described in Materials and Methods.
(TIF)

Figure S12 **LC-ESI-MS negative ion mode spectra of cardiolipin (CA) molecular species of standard preparation.** Arrows indicate different CA molecular species detected. I.S., internal standard; right handed numbers at the top of the Y axis indicate full-scale intensity in arbitrary units. The analysis was carried out as described in Materials and Methods.
(TIF)

Figure S13 **LC-ESI-MS negative ion mode spectra of cardiolipin (CA) molecular species of lipid extract of pellets fraction of** ***E. coli*** **strain BL21DE3-pMAL** ***ptsG*** **.** Arrows indicate different CA molecular species detected in pellet and standard preparations (for standard preparation see Figure S12 at Saier web site). I.S., internal standard; right handed numbers at the top of the Y axis indicate full-scale intensity in arbitrary units. The lipid contents of *E. coli* strain BL21DE3-pMAL*ptsG* were extracted and analyzed as described in Materials and Methods.
(TIF)

Figure S14 **LC-ESI-MS negative ion mode spectra of cardiolipin (CA) molecular species of lipid extract of peak 2 fraction of** ***E. coli*** **strain BL21DE3-pMAL** ***ptsG*** **.** Arrows indicate different CA molecular species detected in peak 2 and standard preparations (for standard preparation see Figure S12 at Saier web site). I.S., internal standard; right handed numbers at the top of the Y axis indicate full-scale intensity in arbitrary units. The lipid contents of *E. coli* strain BL21DE3-pMAL*ptsG* were extracted and analyzed as described in Materials and Methods.
(TIF)
