# Supplementary figures and images for: Biophysical Studies of the Membrane-Embedded and Cytoplasmic Forms of the Glucose-Specific Enzyme II of the E. coli Phosphotransferase System (PTS)
Source: PLoS One. 2011 Sep 15;6(9):e24088. doi: 10.1371/journal.pone.0024088 (PMC3174158; doi:10.1371/journal.pone.0024088)

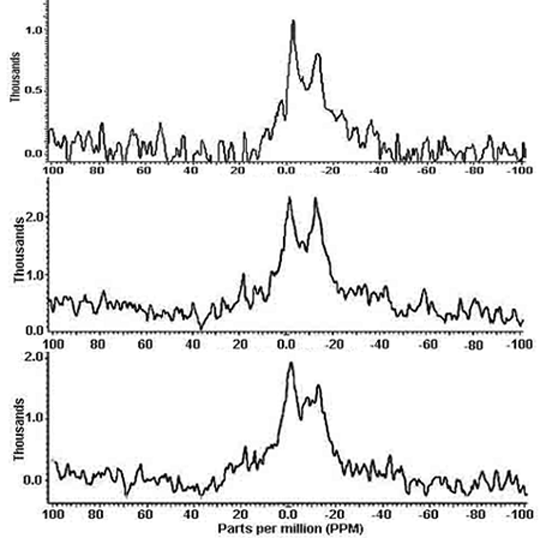

Supplement: Figure S1 — [31P]NMR spectra of purified IIGlc preparations of pellet (Pt) fraction of E. coli strain BW25113Δ ptsG Δ malEKn -pMAL- ptsG . The spectra were recorded at different temperatures (25, 37, 45°C), an acquisition time of 1.6 seconds and 46,000 scans. The sample was exposed sequentially to the applied temperatures. For details see Materials and Methods. (TIF) [file pone.0024088.s001.tif]

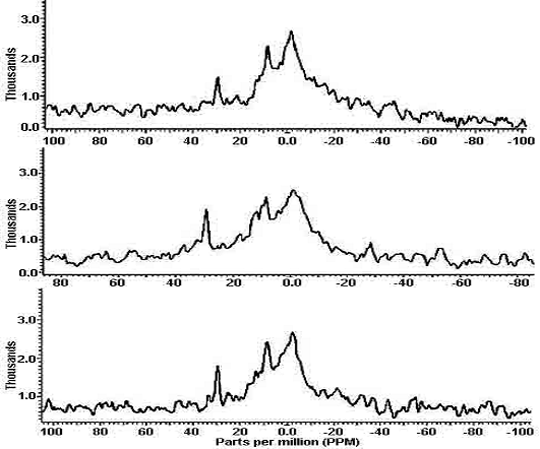

Supplement: Figure S2 — [31P]NMR spectra of purified IIGlc preparation of initial (P1-A) fraction of peak 1 of E. coli strain BW25113Δ ptsG Δ malEKn -pMAL- ptsG . The spectra were recorded at different temperatures (25, 37, 45°C), an acquisition time of 1.6 seconds and 46,000 scans. The sample was exposed sequentially to the applied temperatures. For details see Materials and Methods. (TIF) [file pone.0024088.s002.tif]

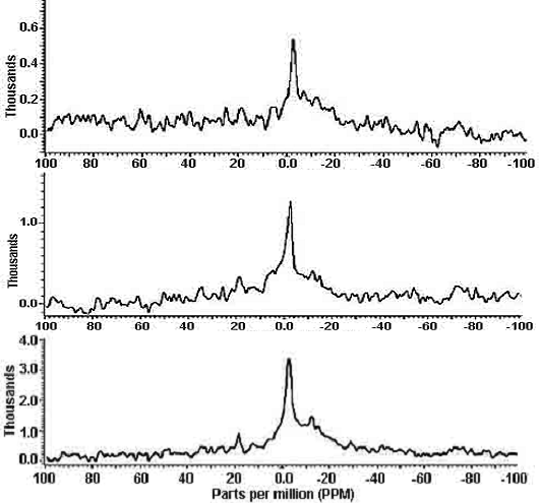

Supplement: Figure S3 — [31P]NMR spectra of purified IIGlc preparation of middle (P1-B) fraction of peak 1 of E. coli strain BW25113Δ ptsG Δ malEKn -pMAL- ptsG . The spectra were recorded at different temperatures (25, 37, 45°C), an acquisition time of 1.6 seconds and 46,000 scans. The sample was exposed sequentially to the applied temperatures. For details see Materials and Methods. (TIF) [file pone.0024088.s003.tif]

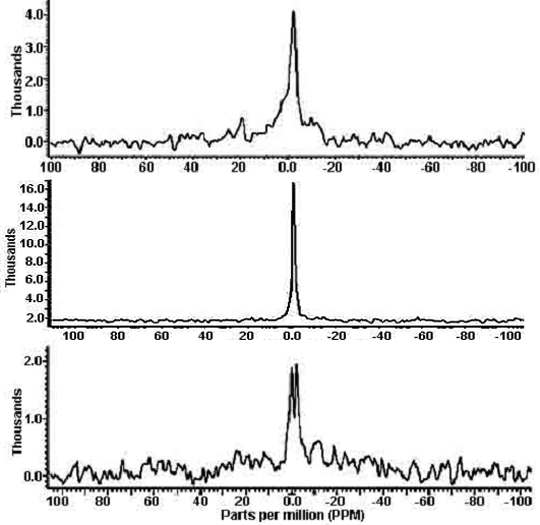

Supplement: Figure S4 — [31P]NMR spectra of purified IIGlc preparation of tail (P1-C) fraction of peak 1 of E. coli strain BW25113Δ ptsG Δ malEKn -pMAL- ptsG . The spectra were recorded at different temperatures (25, 37, 45°C), an acquisition time of 1.6 seconds and 46,000 scans. The sample was exposed sequentially to the applied temperatures. For details see Materials and Methods. (TIF) [file pone.0024088.s004.tif]

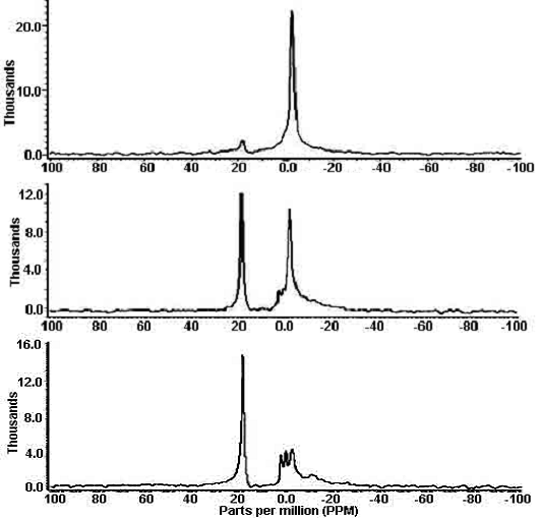

Supplement: Figure S5 — [31P]NMR spectra of purified IIGlc preparation of peak 2 of E. coli strain BW25113Δ ptsG Δ malEKn -pMAL- ptsG . The spectra were recorded at different temperatures (25, 37, 45°C), an acquisition time of 1.6 seconds and 46,000 scans. The sample was exposed sequentially to the applied temperatures. For details see Materials and Methods. (TIF) [file pone.0024088.s005.tif]

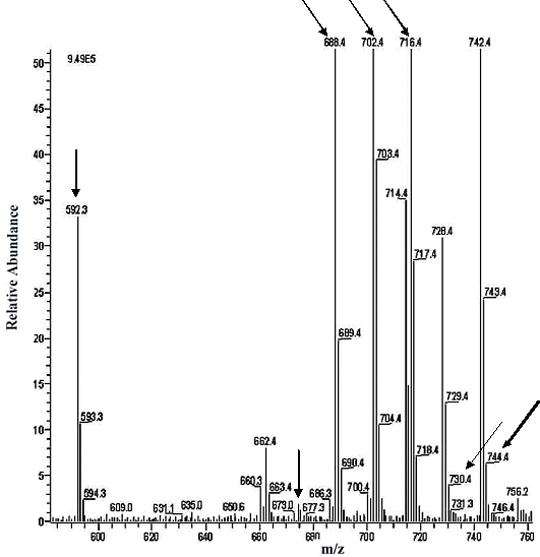

Supplement: Figure S6 — LC-ESI-MS negative ion mode spectra of phosphatidylethanolamine (PE) molecular species in standard preparation. Arrows indicate different PE molecular species detected. I.S., internal standard; right handed numbers at the top of the Y axis indicate full-scale intensity in arbitrary units. The analysis was done as described in Materials and Methods. (TIF) [file pone.0024088.s006.tif]

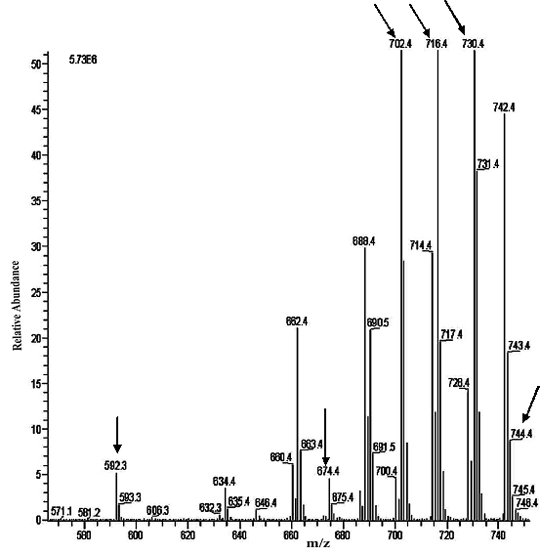

Supplement: Figure S7 — LC-ESI-MS negative ion mode spectra of phosphatidylethanolamine (PE) molecular species of lipid extract of pellet fraction of E. coli strain BL21DE3-pMAL ptsG . Arrows indicate different PE molecular species detected in pellet and standard preparations (for standard preparation see Figure S6 at Saier web site). I.S., internal standard; right handed numbers at the top of the Y axis indicate full-scale intensity in arbitrary units. The lipid contents of E. coli strain BL21DE3-pMALptsG were extracted and analyzed as described in Materials and Methods. (TIF) [file pone.0024088.s007.tif]

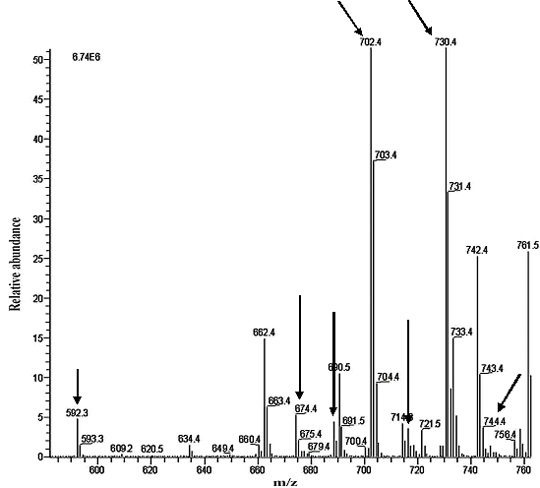

Supplement: Figure S8 — LC-ESI-MS negative ion mode spectra of phosphatidylethanolamine (PE) molecular species of lipid extract of peak 2 fraction of E. coli strain BL21DE3-pMAL ptsG . Arrows indicate different PE molecular species detected in peak 2 and standard preparations (for standard preparation see Figure S6 at Saier web site). I.S., internal standard; right handed numbers at the top of the Y axis indicate full-scale intensity in arbitrary units. The lipid contents of E. coli strain BL21DE3-pMALptsG were extracted and analyzed as described in Materials and Methods. (TIF) [file pone.0024088.s008.tif]

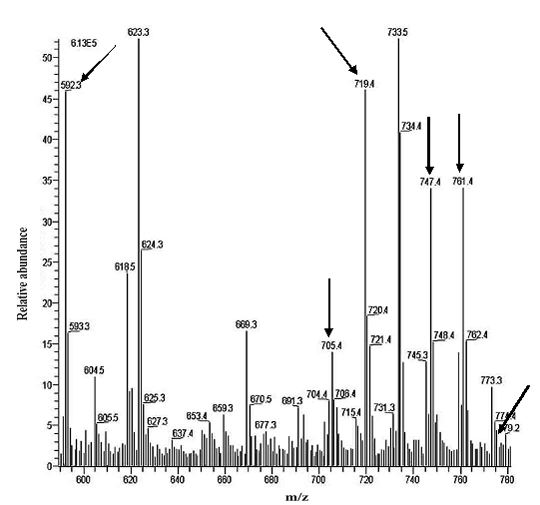

Supplement: Figure S9 — LC-ESI-MS negative ion mode spectra of phosphatidylglycerol (PG) molecular species of standard preparation. Arrows indicate different PG molecular species detected. I.S., internal standard; right handed numbers at the top of the Y axis indicate full-scale intensity in arbitrary units. The analysis was carried out as described in Materials and Methods. (TIF) [file pone.0024088.s009.tif]

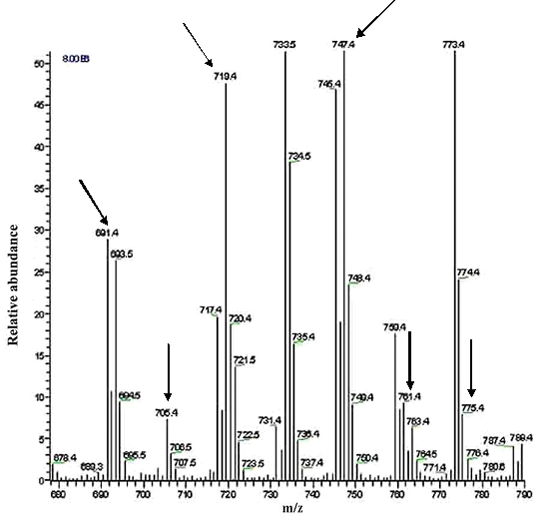

Supplement: Figure S10 — LC-ESI-MS negative ion mode spectra of phosphatidylglycerol (PG) molecular species of lipid extract of pellets fraction of E. coli strain BL21DE3-pMAL ptsG . Arrows indicate different PG molecular species detected in pellet and standard preparations (for standard preparation see Figure S9 at Saier web site). Right handed numbers at the top of the Y axis indicate full-scale intensity in arbitrary units. The lipid contents of E. coli strain BL21DE3-pMALptsG were extracted and analyzed as described in Materials and Methods. (TIF) [file pone.0024088.s010.tif]

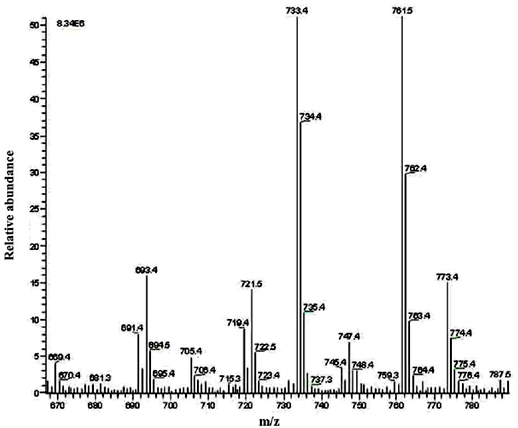

Supplement: Figure S11 — LC-ESI-MS negative ion mode spectra of phosphatidylglycerol (PG) molecular species of lipid extract of peak 2 fraction of E. coli strain BL21DE3-pMAL ptsG . Arrows indicate different PG molecular species detected in peak 2 and standard preparations (for standard preparation see Figure S9 at Saier web site). Right handed numbers at the top of the Y axis indicate full-scale intensity in arbitrary units. The lipid contents of E. coli strain BL21DE3-pMALptsG were extracted and analyzed as described in Materials and Methods. (TIF) [file pone.0024088.s011.tif]

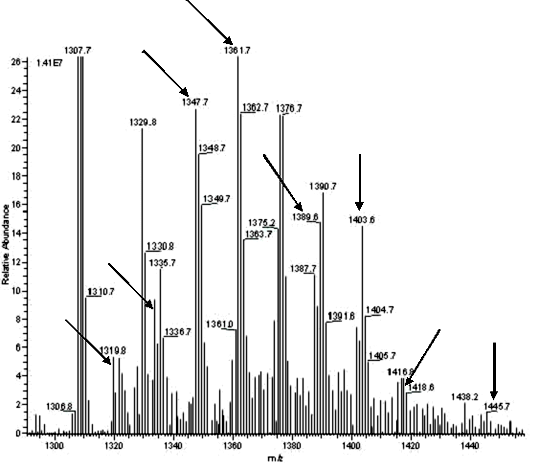

Supplement: Figure S12 — LC-ESI-MS negative ion mode spectra of cardiolipin (CA) molecular species of standard preparation. Arrows indicate different CA molecular species detected. I.S., internal standard; right handed numbers at the top of the Y axis indicate full-scale intensity in arbitrary units. The analysis was carried out as described in Materials and Methods. (TIF) [file pone.0024088.s012.tif]

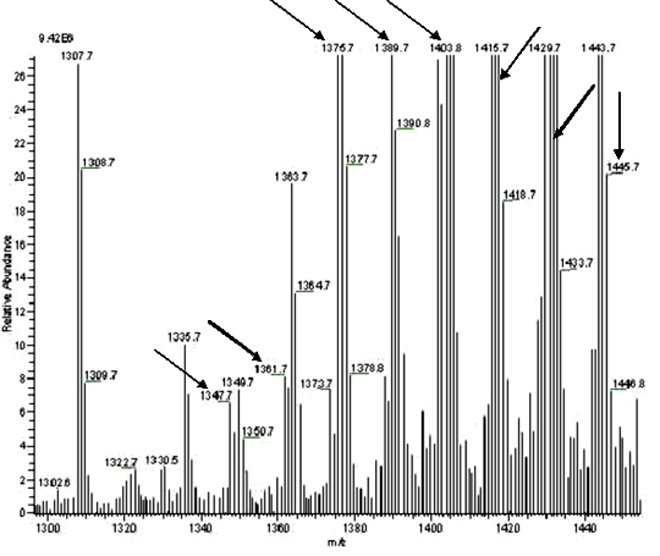

Supplement: Figure S13 — LC-ESI-MS negative ion mode spectra of cardiolipin (CA) molecular species of lipid extract of pellets fraction of E. coli strain BL21DE3-pMAL ptsG . Arrows indicate different CA molecular species detected in pellet and standard preparations (for standard preparation see Figure S12 at Saier web site). I.S., internal standard; right handed numbers at the top of the Y axis indicate full-scale intensity in arbitrary units. The lipid contents of E. coli strain BL21DE3-pMALptsG were extracted and analyzed as described in Materials and Methods. (TIF) [file pone.0024088.s013.tif]

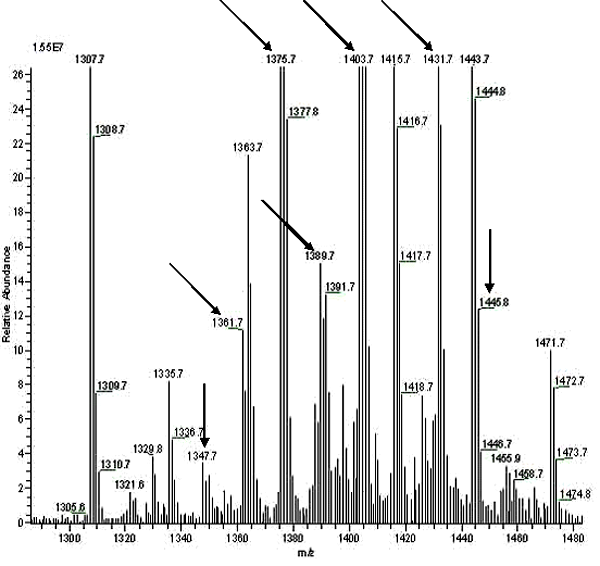

Supplement: Figure S14 — LC-ESI-MS negative ion mode spectra of cardiolipin (CA) molecular species of lipid extract of peak 2 fraction of E. coli strain BL21DE3-pMAL ptsG . Arrows indicate different CA molecular species detected in peak 2 and standard preparations (for standard preparation see Figure S12 at Saier web site). I.S., internal standard; right handed numbers at the top of the Y axis indicate full-scale intensity in arbitrary units. The lipid contents of E. coli strain BL21DE3-pMALptsG were extracted and analyzed as described in Materials and Methods. (TIF) [file pone.0024088.s014.tif]
